# JmjC catalysed histone H2a *N*-methyl arginine demethylation and C4-arginine hydroxylation reveals importance of sequence-reactivity relationships

**DOI:** 10.1038/s42003-024-07183-5

**Published:** 2024-11-27

**Authors:** Joanna Bonnici, Razanne Oueini, Eidarus Salah, Catrine Johansson, Elisabete Pires, Martine Abboud, Robert S. Dawber, Anthony Tumber, Patrick Rabe, Hilal Saraç, Christopher J. Schofield, Akane Kawamura

**Affiliations:** 1 Chemistry Research Laboratory, Department of Chemistry and the Ineos Oxford Institute for Antimicrobial Research, University of Oxford, Oxford, OX1 3TA United Kingdom; 2 Chemistry - School of Natural and Environmental Sciences, Newcastle University, Newcastle Upon Tyne, NE1 7RU United Kingdom; 3 Botnar Research Centre, NIHR Oxford Biomedical Research Unit, University of Oxford, Oxford, OX3 7LD United Kingdom

**Keywords:** Enzyme mechanisms, Chemical modification

## Abstract

2-Oxoglutarate (2OG) dependent *N*^*ε*^-methyl lysine demethylases (JmjC-KDMs) regulate eukaryotic transcription. We report studies showing that isolated forms of all human KDM4 and KDM5 JmjC enzymes catalyse demethylation of *N*-methylated Arg-3 of histone H2a. Unexpectedly, the results reveal that KDM4E and, less efficiently, KDM4D catalyse C-4 hydroxylation of Arg-20 of H2a on peptides, recombinant H2a, and calf histone extracts, including when the Arg-20 guanidino group is *N*-methylated. Combined with previous observations, our biochemical results highlight the importance of sequence context in determining the relative efficiencies of lysine and arginine demethylation reactions catalysed by KDM4s and KDM5s. At least in some cases changes in sequence can also enable a different JmjC reaction mode, such as C-4 arginine hydroxylation instead of demethylation. Further work is thus required to define the full scope of JmjC catalysed reactions in cells.

## Abstract (web summary)

The JmjC histone demethylases KDM4 and KDM5 catalyse demethylation of *N*-methylated Arg-3 of histone H2a, and KDM4E is found − unexpectedly − to catalyse C-4 hydroxylation of Arg-20 of H2a.

## Introduction

**Fig. 1:**
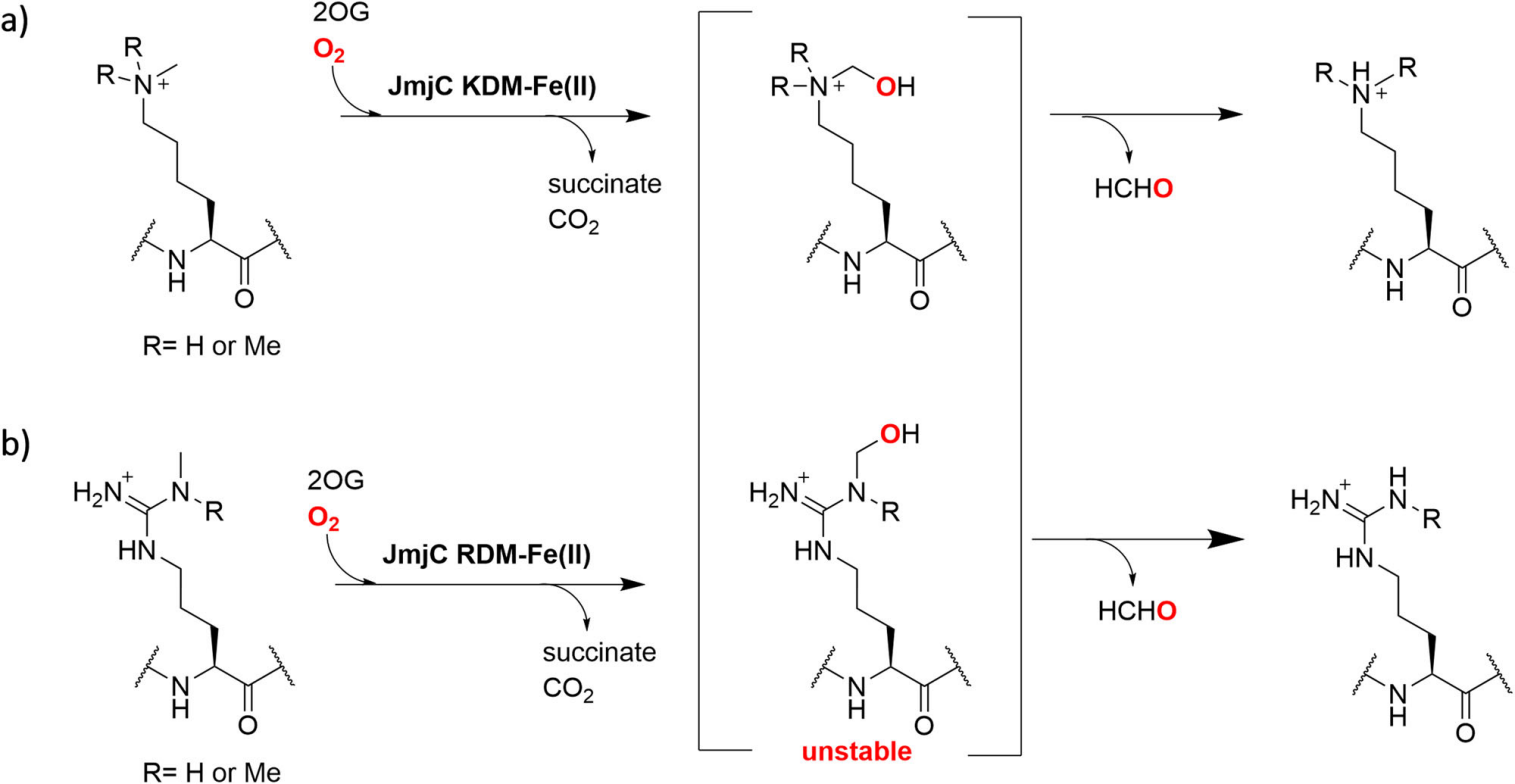
JmjC-KDM catalysed KDM and RDM reactions. JmjC KDM (**a**) / RDM (**b**) catalysed *N*-methyl lysine- and arginine-demethylation occurs via unstable hemiaminal-type intermediates. Note only the mono-methylated (me1) and asymmetric di-methyl (me2a) forms of *N*-methylated Arg are shown. Each 2OG oxygenase catalysed oxidation is coupled to conversion of 2OG / O_2_ to succinate / CO_2_.

The dynamic *N*-methylation and demethylation of lysine- and arginine-residues in histones is important in the regulation of eukaryotic transcription^1–3^. *N*^*ε*^-Methyl lysine demethylation is catalysed by two families of *N*^*ε*^-methyl lysine demethylases (KDMs): the flavin-dependent KDMs (LSDs/KDM1s) and the larger Fe(II) and 2-oxoglutarate (2OG) dependent Jumonji C (JmjC)-KDM family^3,4^. Unlike the KDM1s, the JmjC-KDMs catalyse the demethylation of all tri-, di- and mono- *N*^*ε*^-methyl lysine methylation states via unstable hemiaminal intermediates^5,6^. At least in isolated form, some JmjC-KDMs, including all human KDM5s, also have *N*-methyl arginine demethylation (RDM) activity (Fig. 1)^7,8^. The catalytic JmjC-domains of the JmjC-KDMs are related to those of the JmjC ‘hydroxylases’, most (but not all) of which catalyse hydroxylation of methylene groups of protein residue side chains to give alcohols, which are stable compared to the hemiaminal intermediates in demethylation reactions^3,6,9^. Examples of JmjC hydroxylase catalysed reactions include JMJD5 catalysed C-3 arginine-hydroxylation, and JMJD6 and JMJD7 catalysed lysine-residue C-5 hydroxylation^6,9–14^. Several studies have revealed substrate promiscuity of certain JmjC hydroxylases, in particular for FIH and JMJD6 ^15,16^. There is evidence that the reaction types catalysed by JmjC-KDMs extend beyond the established KDM and less well established RDM activities, i.e. certain *N*^*ε*^-alkylated lysine substrate analogues can be oxidised to form stable alcohols^17^.

The histone H2a *N*-terminal region is less modified than those of histones H3 and H4 (Supplementary Table 1); reported H2a modifications are generally associated with transcriptional repression and may have a role in cancer progression^18,19^. Histones H3, H4 (and to lesser extent H1.4) have been explored as demethylation substrates of the KDM4 and KDM5 families of human JmjC-KDMs, with the results indicating that the preferred sequence contexts for KDM and RDM activities may differ^20–22^. Histone H2a, however, is largely unexplored as a KDM4 and KDM5 substrate.

The observation that some JmjC-KDMs have RDM activity coupled with the knowledge that JmjC ‘hydroxylases’ catalyse a range of sidechain/methylene hydroxylations raises the possibility that some JmjC-KDMs catalyse hydroxylation reactions to give stable alcohols. Here we report studies showing that isolated forms of human KDM4 and KDM5 can catalyse demethylation of *N*-methylated Arg-3 of H2a. Unexpectedly, the results show that KDM4E and, less efficiently, KDM4D both catalyse C-4 hydroxylation of H2a Arg-20. The results provide evidence that the mode of JmjC catalysed reactions can be altered by different substrate sequence contexts.

## Results

### Histone H2a mono- and di-methylated Arg-3 peptides are substrates of KDM5 and KDM4 subfamily members

**Fig. 2:**
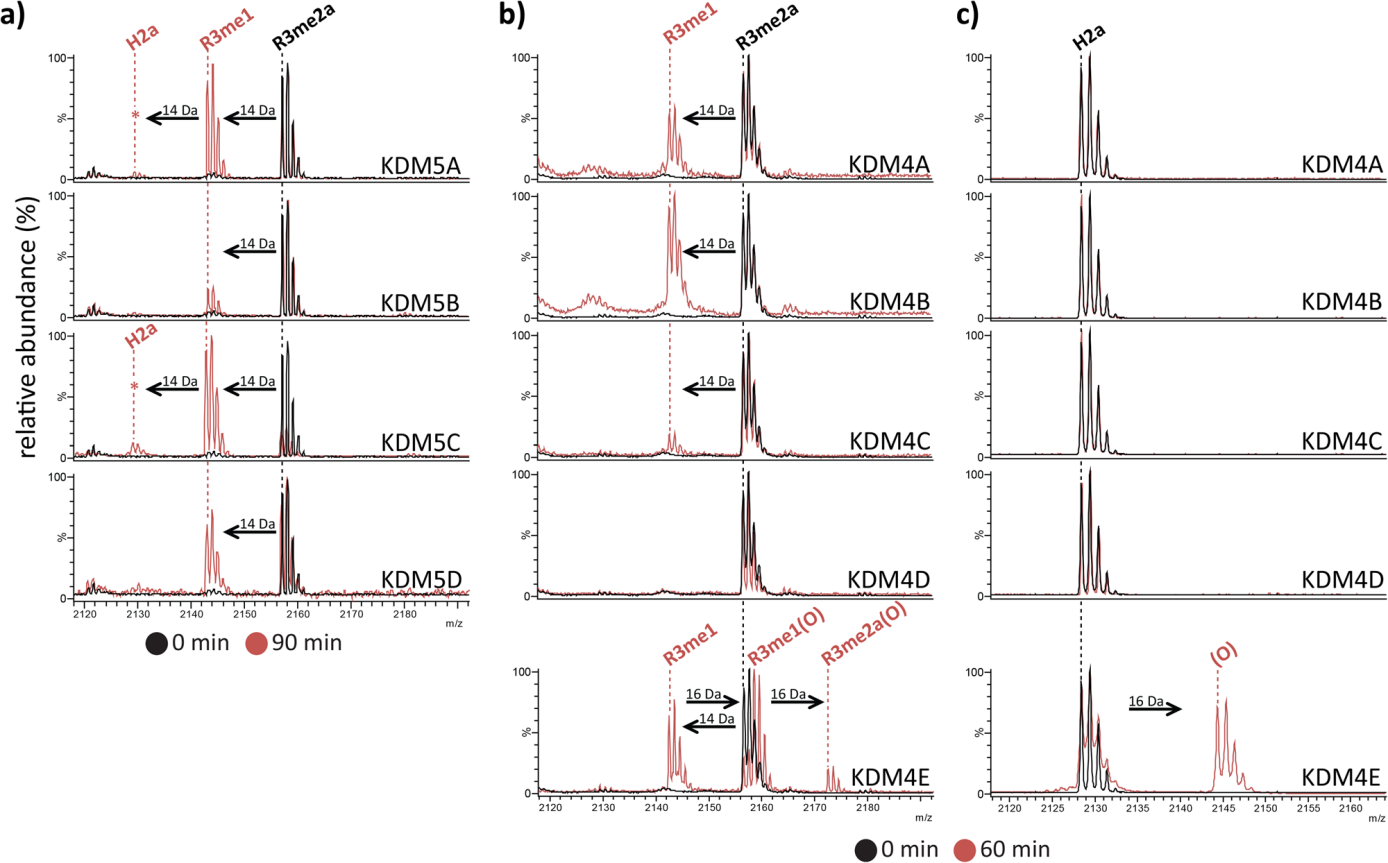
Histone H2a fragments are KDM5 and KDM4 substrates. MS analyses [MH]^+^ reveal −14 Da mass shifts relative to H2a(1–20)R3me2a indicating demethylation on incubation with (**a**) KDM5A-D (90 min, red), (**b**) KDM4A–C/KDM4E (60 min, red). **b**, **c** A +16 Da mass shift occurs with H2a(1–20)R3me2a, H2a(1–20)R3me1 and H2a(1–20) on KDM4E incubation. *Low level demethylation (<10%). See Supplementary Table 6 and Supplementary Fig. 2 for assay conditions and quantification, respectively. Note in the case of KDM4D, low levels of RDM / hydroxylation activity were observed with different assays (Supplementary Figs. 1 and 6). 0-minute MS spectrum in black, 60/90-minute spectrum in red.

To investigate whether histone H2a is a JmjC-KDM substrate, we initially used mass spectrometry (MS) to screen a set of biotinylated *N*-terminal H2a(1–20) peptides with post-translational modifications (PTMs) at Arg-3, i.e. asymmetrically/symmetrically di-methylated Arg-3 (H2aR3me2a, H2aR3me2s, respectively), mono-methylated Arg-3 (H2aR3me1) and citrullinated Arg-3 (H2aR3Ci), against KDM4A, KDM4D, KDM4E and KDM5C. Evidence for RDM activity with H2aR3me2a, H2aR3me2s, and H2aR3me1 was observed with KDM4E and KDM5C, and, to a lesser extent, with KDM4A and KDM4D (Supplementary Fig. 1). H2a(1–20)R3me2a, which appeared to be the most efficient RDM substrate in the screen, was then re-synthesised without a biotin tag and tested for RDM activity using the catalytic domains of all KDM4/KDM5 subfamily members. KDM5A–D all catalysed demethylation of H2a(1–20)R3me2a with similar activity levels as reported for H3(1–21)R2me2a (Fig. 2, Supplementary Fig. 2)^8^. RDM activity was also observed with all KDM4 subfamily members (KDM4A–E), with KDM4E having the highest RDM activity (Fig. 2).

The RDM activity of KDM4E with H2a(1–20)R3me2a was compared with demethylation of H3(1–21)R2me2a using a formaldehyde dehydrogenase (FDH) assay^23,24^. KDM4E catalysed demethylation of H2a R3me2a was less efficient than H3 R2me2a by approximately three-fold under the same conditions (*k*_cat_/*K*_M_ 0.89 hr^−1^ µM^−1^ for H2a(1–20)R3me2a and 2.58 hr^−1^ µM^−1^ for H3(1–21)R2me2a). KDM4E had a four-fold higher affinity for H2a(1–20)R3me2a (*K*_M_ 5.18 µM) than for H3(1–21)R2me2a (*K*_M_ 20.69 µM) (Supplementary Fig. 3). Of note, the *k*_cat_/*K*_M_ values for RDM substrates for KDM4E were lower than for its cognate KDM substrate H3 K9me3, using the same FDH assay (H2a R3me2a and H3 R2me2a were 39-fold and 13-fold less than H3 K9me3 respectively)^7^.

In general, KDM5 RDM activity with H2a(1–20)R3me2a was higher than that with KDM4E (KDM5C was 2.5 times more active than KDM4E; specific activities: 0.37 pmol min^−1^ µM^−1^ and 0.15 pmol min^−1^ µM^−1^_,_ respectively) (Supplementary Fig. 4). Full length KDM4A has similar RDM activity with H2a(1–20)R3me2a as observed for the KDM4A catalytic domain (Supplementary Fig. 5).

### KDM4E catalyses the C-4 hydroxylation of histone H2a Arg-20

In addition to RDM activity, a new +16 Da mass peak consistent with hydroxylation, was observed with almost all of the H2a(1–20) peptides screened with KDM4D and KDM4E, but not with KDM4A nor KDM5C. With KDM4E, +16 Da mass shifts were observed for all biotin tagged H2a peptides (Supplementary Fig. 1); H2a(1–20), which gave a single apparent product, was chosen for further investigation. H2a(1–20) was re-synthesised without a biotin tag (which can undergo S-oxidation) and tested for activity against KDM4A–E. A single +16 Da product peak indicating apparent hydroxylation was observed (~42% conversion) with KDM4E (Fig. 2c); a lower level of apparent hydroxylation was observed with KDM4D (Supplementary Fig. 6); no evidence for KDM4A-C catalysed hydroxylation was observed (Fig. 2, Supplementary Fig. 1). Note that in the MS assays, the mixture of products resulting from KDM4E catalysed demethylation of H2a(1–20)R3me2a and H2a(1–20)R3me1 and hydroxylation of H2a(1–20)R3me2a, H2a(1–20)R3me1, and H2a(1–20) (Fig. 2), complicates detailed kinetic analysis.

**Fig. 3:**
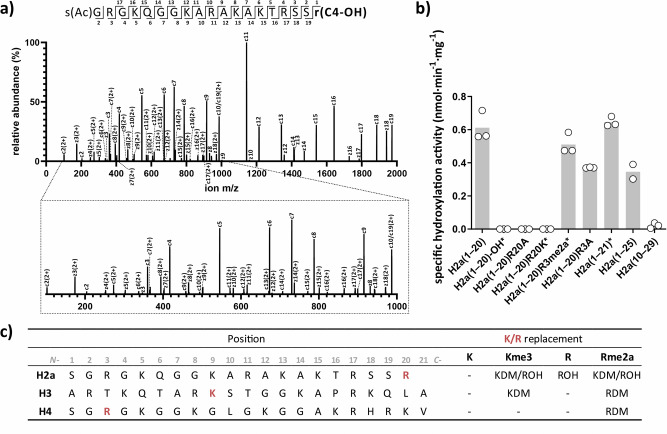
KDM4E-catalysed hydroxylation occurs at Arg-20 on histone H2a peptide. **a** LC–MS/MS analysis of H2a(1–20) after KDM4E treatment. Two potential hydroxylation sites, R17 and R20, were identified, with the latter more likely (Supplementary Fig. 9). **b** Peptide variant studies support Arg-20 hydroxylation; specific activities: H2a(1–20), 0.65 nmol min^−1^ mg^−1^ SD: 0.073; H2a(1–20) with a C-terminal carboxylic acid (indicated with an -OH), no activity; H2a(1–20)R20A, no activity; H2a(1–20)R20K*, no activity; H2a(1–20)R20Kme3*, 0.44 nmol min^−1^ mg^−1^ SD: 0.04; H2a(1–20)R3me2a*, 0.51 nmol min^−1^ mg^−1^ SD: 0.05; H2a(1–20)R3A, 0.37 nmol min^−1^ mg^−1^ SD: 0.002; H2a(1–21)*, 0.65 nmol min^−1^ mg^−1^ SD 0.02; H2a(1–25, 0.35 nmol min^−1^ mg^−1^ SD: 0.044; and H2a(10–29), 0.02 nmol min^−1^ mg^−1^ SD: 0.013. Measurements employed MALDI–TOF MS except when labelled with*, which employed LC–MS (Supplementary Fig. 10 for representative MS data). *n* = 3 independent assays. **c** KDM, RDM and hydroxylation (+16 Da) activities of KDM4E are sequence and context dependent. Histone peptides (H2a, H3, H4) and variants (wild type residue in red, replaced with either K, Kme3, R or Rme2a) were incubated with KDM4E and analysed on MALDI–TOF MS. KDM, RDM and hydroxylation (+16 Da) were detected in some but not all peptides (Supplementary Fig. 10 for representative MS data). (−) No mass changes observed. *n* = 3 independent assays. See Supplementary Table 6 for conditions.

**Fig. 4:**
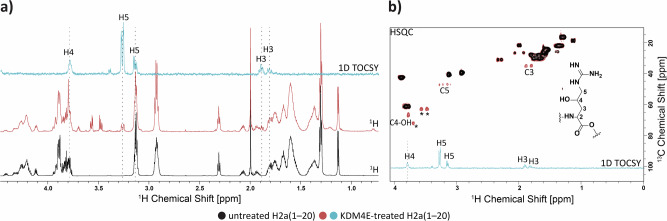
Arginine hydroxylation by KDM4E occurs at C-4 of H2a Arg-20. NMR analyses of unmodified and KDM4E-treated H2a(1–20). (**a**) ^1^H NMR spectra of unmodified (black) and hydroxylated H2a (red). Note the new multiplet at ~3.2 ppm in the latter (blue), assigned as one of the 5-Hs of C-4 hydroxylated R20. (**b**) Overlay of HSQC spectra of unmodified (black) and hydroxylated H2a (red) and 1D TOCSY spectrum of the hydroxylated H2a (irradiation at H5); the peak assigned as hydroxylated C-4 is at ^1^H 3.8 ppm, ^13^C: 67 ppm. *Residual buffer and glycerol. Supplementary Fig. 11 is a ^1^H-^1^H COSY spectrum of hydroxylated H2a(1–20).

To compare the efficiencies of KDM4E catalysed demethylation and hydroxylation, H2a(1–20) substrate depletion and product formation were monitored by assays using liquid chromatography (LC) coupled to MS; the specificity constant (*k*_cat_/*K*_M_) for hydroxylation was in a similar range (*k*_cat_/*K*_M_ 0.77 hr^−1^ µM^−1^) to that of demethylation of H2a(1–20)R3me2a (Supplementary Fig. 3). Note, different methods (FDH for RDM and LC–MS for hydroxylation) were used for the assays, hence caution should be taken in comparing these values. Formation of the +16 Da product obtained by KDM4E catalysed oxidation of H2a(1–20) was 2OG and O_2_ dependent, and was inhibited by broad spectrum 2OG oxygenase inhibitors [IOX1, 2,4-pyridine-2,4-dicarboxylate (2,4-PDCA), *N*-oxalylglycine (NOG)] (Supplementary Figs. 7 and 8). A +18 Da mass shift relative to H2a(1–21) was observed when the reaction was carried out under ^18^O_2_, while a +16 Da mass shift was observed when the reaction was carried out in H_2_^18^O; these observations demonstrate that hydroxylation results from oxygenase catalysis (Supplementary Fig. 8). LC–MS/MS fragmentation analyses on the H2a(1–20) +16 Da product of KDM4E catalysis revealed two possible hydroxylation sites, i.e., R17 (Supplementary Fig. 9) and R20 (Fig. 3a), with the latter appearing more likely, as supported by a lack of a +16 Da mass shift on incubation with H2a(1–20)R20A (Fig. 3b, Supplementary Fig. 10b). NMR analysis revealed the site of hydroxylation on H2a R20 to be C-4, likely as a result of formation of a single C-4 epimer, though we were unable to assign its stereochemistry (Fig. 4, Supplementary Fig. 11).

To investigate whether hydroxylation occurs when a potential substrate arginine-residue is not at the C-terminus, KDM4E was incubated with other H2a fragments. The extent of hydroxylation with H2a(1–21) was similar to that with H2a(1–20), but was lower for H2a(1–25), though a +16 Da mass shift was still clearly observed. For H2a(10–29), the activity was diminished indicating the *N*-terminal H2a region is important for hydroxylation. Consistent with this, the H2a(1–20)R3A variant manifested reduced activity relative to H2a(1–20), though substantial reduction in hydroxylation activity was not observed with H2a(1–20)R3me2a. When the C-terminal amide of H2a(1–20) was substituted for a carboxylic acid (H2a(1–20)-OH), no activity was observed. No evidence for hydroxylation was observed when Arg-20 was replaced with Lys (H2a(1–20)R20K) (Fig. 3b, Supplementary Fig. 10).

Peaks corresponding to both hydroxylation and demethylation were observed when Arg-20 was substituted with tri-methylated lysine (H2a(1–20)R20Kme3). Notably, H2a(1–20)R20Kme3 was apparently hydroxylated revealing another reaction mode (i.e. tri-methyl lysine-residue hydroxylation). Masses corresponding to the hydroxylated, demethylated products H2a(1–20)R20Kme1 and H2a(1–20)R20Kme2 were observed, though the timing(s) at which hydroxylation(s) occurs is unclear because the hydroxylated products can undergo demethylation (Fig. 3c, Supplementary Fig. 10d).

With H2a(1–20)R20me2a, H2a(1–20)R20me2s and H2a(1–20)R20me1, in addition to peaks corresponding to demethylated and hydroxylated products, −2 Da peaks, relative to H2a(1–20)R20me2s, H2a(1–20)R20me1 and H2a(1–20)R20me2s(C4-OH), but not to H2a(1–20)R20me2a, were observed. The −2 Da peaks were more pronounced after 16-hour incubation. We have not determined the structures giving rise to the −2 Da peaks; they may reflect low levels of desaturation of the Arg-20 methylenes to form an alkene(s), as precedented in reactions with other 2OG oxygenases acting on small molecules and (with some substrates) the JmjC protein hydroxylase FIH (Fig. 3c, Supplementary Fig. 12)^6,25,26^.

Interestingly, the results imply that whether KDM4E acts as an *N*-methyl lysine demethylase (KDM), an *N*-methyl arginine demethylase (RDM), or a hydroxylase, can be substrate sequence context dependent. At the R20 position in H2a, KDM4E can act as a KDM and an RDM, as well as a hydroxylase (Fig. 3b, c, Supplementary Fig. 10). However, when the K9 position of histone H3 is *N*-methylated (Kme3 / Rme2a) or replaced with an unmodified Arg-residue, only KDM and RDM activities are observed (Fig. 3c, Supplementary Fig. 10). At the R3 position of histone H4 we saw no evidence for hydroxylase activity and when R3 was *N*-methylated (Kme3 / Rme2a), only RDM activity is observed (Fig. 3c, Supplementary Fig. 10).

With H2a(1–20)R20me2a or H2a(1–20)R20me2s and KDM4D, low levels of potential RDM activity were observed with both peptide fragments; there was no evidence for hydroxylation with H2a(1–20)R20me2a and a low level potential hydroxylation with H2a(1–20)R20me2s. This level of activity is comparable with that achieved with KDM4D and H2a(1–20)R3me2a or H2a(1–20). No activity was observed with KDM4D and H2a(1–20)R20Kme3 (Supplementary Fig. 13).

**Fig. 5:**
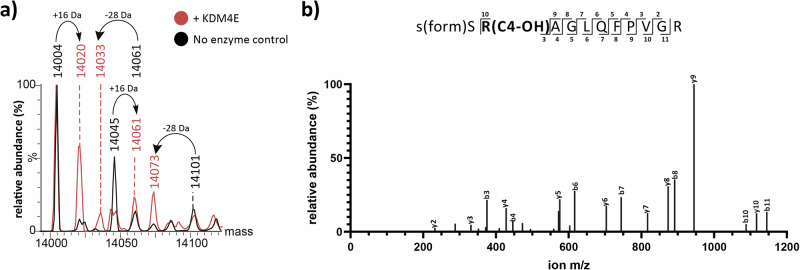
KDM4E catalyses Arg-20 hydroxylation of recombinant histone H2a and calf thymus histone H2a. **a** Representative deconvoluted LC–MS analysis of KDM4E-treated calf histone H2a (red) showing a +16 Da mass shift corresponding to addition of a hydroxyl group and −28 Da mass shifts corresponding to demethylation. Three independent assay repeats were carried out. ‘No enzyme control’ spectrum in black, ‘KDM4E-treated sample spectrum in red. **b** LC–MS/MS analysis of KDM4E-treated calf histone H2a. No evidence for hydroxylation was detected in the S(+27.99)SRAGLQFPVGR (−10logP: 50.3) fragment from the starting material (See Supplementary Fig. 15a) while evidence for hydroxylation at R20 was observed in the corresponding fragment with KDM4E treatment, i.e. (−10logP: 32.2) S(+27.99)SR(+16.00)AGLQFPVGR. See Supplementary Fig. 15b,c for ion tables. The +28 Da mass shift on the *N*-terminal Ser is due to *N*-formylation caused by formic acid used to quench the tryptic digestion.

To investigate whether hydroxylation can occur on a histone protein, KDM4E was incubated with recombinant H2a. MS fragmentation studies provided evidence that the site of this +16 Da mass shift is at H2a R20 (Supplementary Fig. 14). When calf histone extracts were incubated with isolated KDM4E, mass shifts corresponding to two demethylations (−28 Da) and hydroxylation (+16 Da) were observed (Fig. 5a), with H2a R20 being observed to be hydroxylated by LC–MS/MS analysis (Fig. 5b, Supplementary Fig. 15).

## Discussion

The combined studies clearly demonstrate that the relative efficiency of KDM and RDM reactions catalysed by JmjC-KDMs can vary depending on the sequence context (Fig. 3c, Supplementary Fig. 1, Supplementary Table 2). Strikingly, all the subfamily members of human KDM4s and KDM5s catalysed demethylation of H2a(1–20)R3me2a (Fig. 2). Previous studies have shown RDM activity for all KDM5s with H3 R2me2a, but whether all KDM4s have RDM activity has been less clear, with the highest level of RDM activity being observed with KDM4E and lower levels/no activity being observed with KDM4A–D when using the same substrates^7,8^. The clear RDM activity for all of KDM4A-E with H2a(1–20)R3me2a reported here increases the likelihood of biological relevance of KDM4 subfamily RDM activity. It should be noted that in the chromatin environment in cells, other precedented factors may be important in regulating JmjC-KDM / RDM activity, including other PTMs and / or binding interactions that may target a particular substrate residue to a JmjC active site^27–30^.

**Fig. 6:**
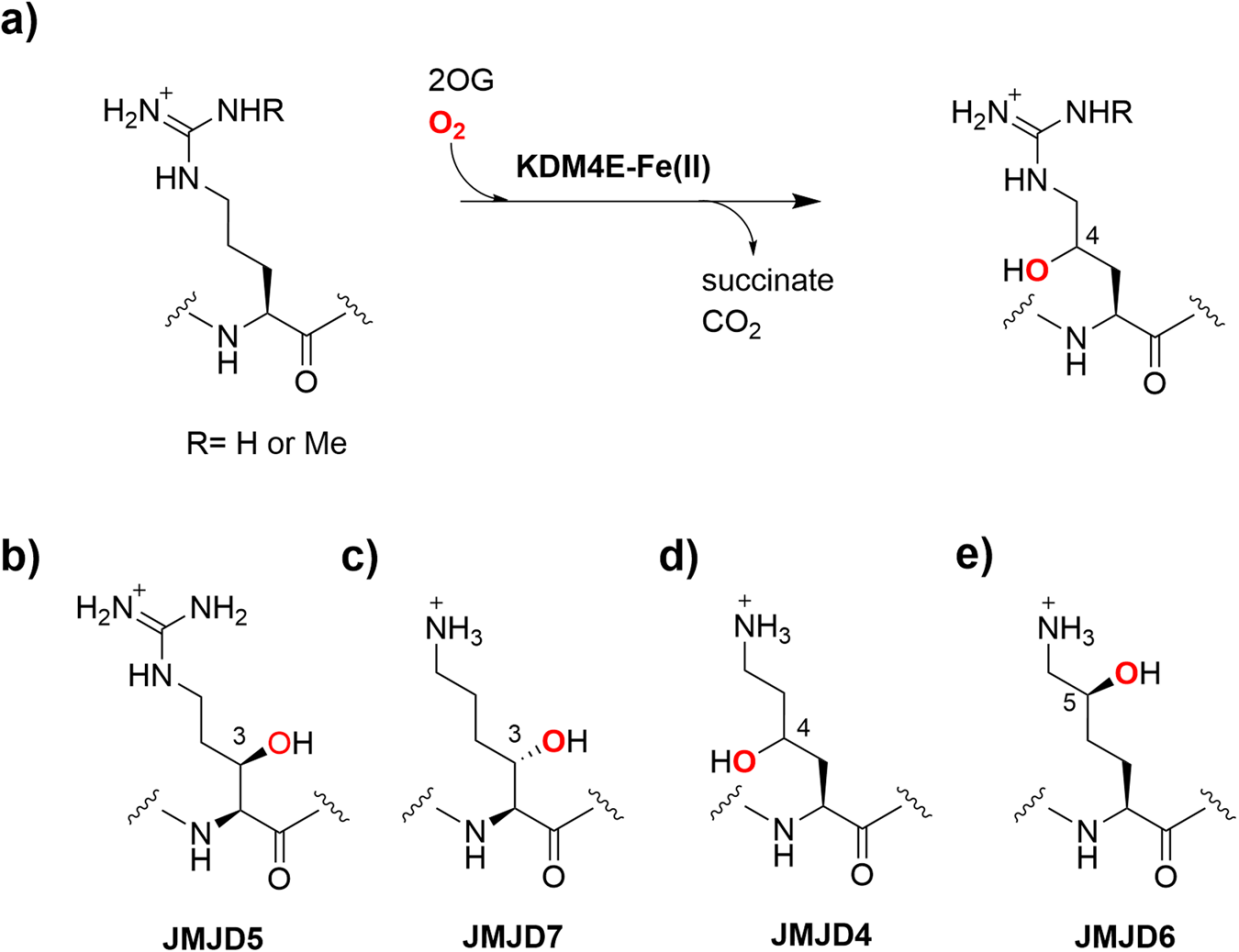
KDM4E-catalysed C-4 arginine hydroxylation and currently described forms of lysyl- and arginyl-hydroxylation catalysed by the 2OG oxygenases JMJD4–7. **a** KDM4E catalysed C-4 hydroxylation of an arginine residue. **b** 3*R*-Hydroxyarginine formation catalysed by JMJD5^13^, (**c**) 3*S*-Hydroxylysine formation catalysed by JMJD7^69^, (**d**) 4-Hydroxylysine formation catalysed by JMJD4^70^, and **e** 5*S*-Hydroxylysine formation catalysed by JMJD6^71–73^. Note these modifications are thermodynamically stable compared to the hemiaminal intermediates involved in demethylation (see Fig. 1).

Unexpectedly, our results reveal that KDM4E and, less efficiently, KDM4D catalyse C-4 hydroxylation of Arg-20 of H2a. In the case of KDM4E this was shown to occur at C-4 in an O_2_ and 2OG-dependent manner, and the reaction was inhibited by broad -spectrum 2OG oxygenase inhibitors (Supplementary Figs. 7 and 8). Hydroxylation was observed whether or not the guanidino group of H2a R20 was *N*-methylated, and to occur both in H2a peptide fragments and H2a protein including from calf thymus (Figs. 3, 5 and 6a).

When H2a Arg-20 is replaced with tri-methylated lysine H2a(1–20)R20Kme3, the demethylated products (Kme2, Kme1, K) were also observed to be hydroxylated, likely at the lysine-residue. Mass spectrometric evidence was also observed for desaturation (at a lower level than hydroxylation) of H2a substrates only when H2a R20 was methylated (Rme2s, Rme1). Although not biologically relevant, from a mechanistic perspective it is interesting that evidence for both hydroxylation and demethylation of the same methylated H2a substrate (Arg-20) occurs, revealing the possibility of two reaction modes on the same residue (though hydroxylation was not observed with H2a(1–20)R20K).

Studies with varied H2a peptides suggest a role for residues at the *N*-terminal region in regulating KDM4E catalysed hydroxylation at R20. Deletion of residues 1–9 led to substantial loss of KDM4E catalysed R20 hydroxylation activity and the H2a R3A substitution had a stronger diminutive effect on hydroxylation compared to demethylation at R3 (Fig. 3b, Supplementary Fig. 10e, f, i). Exactly how the substrate sequence influences the efficiency and type of JmjC ‘KDM’ reaction is unclear. Comparison of KDM4 substrate sequences does not reveal clear differences relating to the different experimentally observed substrate and product selectivities (Supplementary Table 2). Further, there are only a limited number of JmjC KDM4 substrate complex structures reported to date, and as yet we do not have structures of a KDM4 with an H2a fragment^7,22,31–38^. Studies on the H3 K9me3 versus H3 K36me3 selectivity of KDM4 subfamily members suggest multiple interactions are involved^36^.

Whilst KDM and RDM catalysis appear to proceed via broadly similar (though not identical) binding modes for the *N*-methyl lysine and arginine side chains (if not necessarily the rest of the substrate)^7,31,34^, it is difficult to envisage how this can be the case for C-4 arginine hydroxylation. A productive binding mode for C-4 hydroxylation (and maybe desaturation) might be achieved by a turn in the arginine side chain centred at C-3/C-4, possibly accompanied by a different orientation of the ferryl intermediate to that enabling KDM / RDM catalysis (Figs. 1 and 6a). Modelling studies may be informative in exploring the different possible substrate binding modes at KDM active sites and in understanding how different sequences relate to different reaction modes^39^.

The observation of JmjC-KDM catalysed H2a R20 hydroxylation expands the scope of human JmjC catalysis. In microorganisms and plants, 2OG oxygenases catalyse a broad range of oxidative reactions^40,41^. In some cases, the same 2OG oxygenase catalyses different types of oxidative reactions, e.g. clavaminic acid synthase catalyses hydroxylation, oxidative cyclisation, and desaturation reaction at the same active site^42^. In animals, the current evidence is that the scope of 2OG oxygenase reactions is more limited, though some animal 2OG oxygenases accept multiple substrates as exemplified by some JmjC-KDMs and the JmjC hydroxylases FIH and JMJD6^6,9,14,15,20,22,43–49^. Certain *N*^*ε*^-alkyl lysine substrate analogues have been shown to be hydroxylated by JmjC-KDMs to give stable alcohol products^7,17^.

Although C-4 hydroxy arginine has not been reported in animal proteins, arginine C-4 hydroxylation occurs in animal-derived peptides, e.g. (2*S*,3*S*,4 *R*)-3,4-di-hydroxyarginine from a sponge^50^, in adhesive plaques produced by blue mussels^51^, and has been isolated from lentil seeds^52^. C-4 Hydroxy citrulline occurs in antibacterial peptides from *Streptomyces sp*., where it is produced by 2OG oxygenase catalysis^53,54^, and a C-4 hydroxylated arginine is present at the active site of a carbon monoxide dehydrogenase in *Hydrogenophaga pseudoflava*^55^. The 2OG oxygenase JMJD5 catalyses C-3 arginine residue hydroxylation and JMJD7, JMJD4 and JMJD6 catalyse C-3, C-4 and C-5 lysine-residue hydroxylation respectively (Fig. 6)^6,9–14^. It thus seems quite possible that C-4 hydroxy arginine will occur in animal proteins and future work can search for it in the expanding set of human proteomic datasets.

The biological role, if any, of H2a R20 hydroxylation is unclear and relatively little is known about the roles of histone H2a. H2a R20 is conserved (when absent being replaced by lysine) and is located after the conserved H2a repression (HAR) domain, a region linked to breast cancer (Supplementary Table 3)^56^. The T16S H2a variant (H2aC1) is overexpressed in oestrogen dependent tumour cells and oestrogen receptor positive, but not negative, cells; the H2aC1 HAR domain is reported to recruit the oestrogen receptor to the promoter regions of BCL2 and c-MYC^18,57^. One possibility is that H2a Arg-20 hydroxylation affects HAR-domain-mediated transcriptional regulation.

KDM4E has been proposed to be a pseudogene^21^, though subsequent work has reported that it is expressed at low levels in specific tissues (at relatively high levels in the testes^58,59^) and cell contexts, e.g., in embryonic development^60–62^ and in neuroblastoma with poor prognosis^63^. The observation of H2a hydroxylation at R20 as catalysed by KDM4E, adds further complexity to its potential roles as a KDM and RDM. Our results highlight both the complexity of chromatin modifications and the need for tools to enable the confident detection of histone post-translational modifications in vivo.

Given the established role of 2OG oxygenases in sensing, in particular hypoxia /O_2_ sensing^64^, in future work it will be of interest to study if variations in O_2_ concentrations affects substrate, or perhaps less likely, product selectivity. Work has shown that, at least some 2OG oxygenases can accept 2-oxo acids other than 2OG as co-substrates^65,66^. Since different binding modes are likely required for demethylation versus hydroxylation, it will be of interest to test if alternative co-substrates can alter KDM4 product selectivity.

The overall results further demonstrate the capacity of 2OG oxygenases and, in particular JmjC enzymes, to catalyse different types of protein oxidation. Although our work has focussed on KDM4s / KDM5s, it would seem likely that other JmjC enzymes will catalyse unexpected reactions. It is important to note that, despite efforts, we have not yet been able to demonstrate that H2a R20 hydroxylation occurs in cells; indeed, definitive demonstration of native hydroxylation in vivo can be challenging^67^. We hope our results will enable work by others to search for arginine hydroxylation, desaturation, and related post-translational modifications in histones and other proteins.

## Methods

### Materials

Unless otherwise stated, reagents and solvents were from Merck-Sigma Aldrich. Enzymes were recombinantly expressed and purified as reported^8^. See Supplementary Table 4 for protein information, purity and masses.

### Peptide synthesis and purification

Peptides made in-house were synthesised using either a Liberty Blue machine (CEM) or a PurePep Chorus (Gyros Protein Technologies) and were HPLC purified to a >95% purity, according to reported procedures^8^. See Supplementary Table 5 for the list of peptides used.

### Enzyme assays

MS-based screening assays with peptides and calf thymus histones were as reported^8^; see Supplementary Table 6 for conditions. Time course assays were based on reported procedures^24^. In brief, equal volumes of the enzyme and substrate solutions were combined to initiate the reaction; 10 µL of 2% (v/v) formic acid (HCOOH) in ddH_2_O or 100% methanol was added to 10 µL of the reaction mixture to quench reactions at different time points. Unless otherwise stated, time course assays were carried out in triplicate. In some cases, LC–MS was used instead of MALDI-TOF MS to avoid a MALDI matrix induced artefact with the hydroxylated product (Supplementary Fig. 16). These two methods were confirmed to produce comparable results (Supplementary Fig. 17).

### Specific activity assays

Data collected from time course assay(s) were used to plot the % conversion vs time (min) using GraphPad Prism5 version 5.04; the resultant slopes were used to fit a line with linear regression to the first-order region of the reaction. The resultant gradient was then converted from % product formation min^−1^ to amount of peptide converted (demethylation and / or hydroxylation) in µM. The mean specific activities and SD errors derived from these data sets are given in mol min^−1^ mg^−1^.

### Michaelis–Menten kinetics

When LC–MS-based assays were used, time course assays were carried out as described^8^; product formation (%) was plotted against time (s) using GraphPad Prism5 version 5.04. The gradient of each reaction was calculated by fitting a line of linear regression to the first-order region. The resultant slope was used to calculate reaction rate in µM hr^−1^. The rate versus peptide concentration (µM) was plotted and the *K*_M_, *k*_cat_, and V_max_ values determined by fitting the Michaelis–Menten equation using GraphPad Prism5 version 5.04. When a formaldehyde dehydrogenase coupled (FDH) based enzyme activity assay was used, analyses were carried out as above and as previously detailed^8^; linear regression was fitted to the first order region of a graph of relative fluorescent units (RFU) vs time (s).

### Inhibition assays

Inhibition of KDM4E catalysed hydroxylation of H2a was investigated using broad-spectrum 2OG mimetics (2, 4-PDCA, NOG, and IOX1). All compounds were dry dispensed into a 384-well polypropylene plate using an ECHO 550 acoustic dispenser (Labcyte, Sunnyvale, CA) to produce a 12-point concentration series with a 2-fold dilution (100–0.05 µM). KDM4E (2 µM) was dispensed across the plate using a Thermo multidrop reagent dispenser (5 µl per well). KDM4E was pre-incubated with the inhibitors for 15 minutes and then the reaction started by dispensing 5 µl of substrate mix per well (200 µM sodium L-ascorbate, 20 µM (NH_4_)_2_Fe(SO_4_)_2_, 200 µM 2OG, 20 µM H2a(1–20) peptide substrate). The enzyme reaction was progressed for 90 minutes and stopped by addition of 10 µL of 2% (v/v) HCOOH. The reaction samples were then analysed by LC–MS using an Agilent 1290 infinity II LC system connected to an Agilent 6550 accurate mass iFunnel quadrupole-time-of-flight (QTOF) machine. The sample (2 µL) was injected onto a ZORBAX RRHD Eclipse Plus C18 column (Agilent); all flow rates were set at 0.2 mL/min. The mobile phase solvent A comprised 100% LC–MS grade water containing 1% (v/v) LC–MS grade HCOOH. Mobile phase solvent B comprised 100% Acetonitrile containing 1% (v/v) LC–MS grade HCOOH. Peptide was separated from enzyme and small molecule using a step wise gradient (0 min–0% solvent B, 4 min–0% solvent B, 7 min–30% solvent B, 8 min–95% solvent B, 9 min–95% solvent B, 10 min 5% solvent B). The column was then re-equilibrated with a 1.5-minute post-run with 95% solvent A. The mass spectrometer was operated in the positive electrospray ionisation (ESI) mode with a nitrogen drying gas temperature (280 °C), drying gas flow rate (13 L/min), nebuliser pressure (40 psig), sheath gas temperature (350 °C), sheath gas flow rate (12 L/min), capillary voltage (4000 V), nozzle voltage (1000 V), fragmentor voltage (365 V). Acquired data were analysed using Masshunter software (Agilent, version B.07.00). The predominant charge state (+5) for the non-hydroxylated and hydroxylated peptides was extracted and integrated when extracted. % hydroxylation was calculated using excel and curves generated using GraphPad Prism (Version 5.04).

### Labelling experiments with ^18^O_2_ and ^18^O-water

Labelling experiments on KDM4E with H2a(1–21) were conducted under controlled ^16^O_2_ or ^18^O_2_ (97 ATOM-% ^18^O, Merck, US) conditions using a Schlenk line setup. Before the experiments, all solutions and solids were transferred into an anaerobic chamber (Belle Technology, O_2_ concentration <2 ppm) and equilibrated overnight. Stock solutions of sodium L-ascorbate (100 mM) and 2OG (100 mM) were prepared in ddH_2_O, and (NH_4_)_2_Fe(SO_4_)_2_ (400 mM) in 20 mM HCl. These solutions were then diluted to final working concentrations of sodium L-ascorbate (10 mM), 2OG (20 mM) in HEPES buffer (50 mM in LC–MS grade H_2_O, pH 7.5), and (NH_4_)_2_Fe(SO_4_)_2_ (1 mM in ddH_2_O). The final sample (200 µL) contained KDM4E (10 µM), H2a(1–21) (20 µM), sodium ascorbate (100 µM), 2OG (200 µM), and (NH_4_)_2_Fe(SO_4_)_2_ (20 µM).

The anaerobic sample was placed into a gas-tight customised 96-well plate holder with a gas inlet. This setup was removed from the anaerobic chamber and connected to the Schlenk line, which was attached to either ^16^O_2_ or ^18^O_2_. Residual O_2_ was purged from the system with argon and vacuum cycles. To create a mild vacuum in the plate holder, the pressure was adjusted to 700 mbar, and the gas inlet was opened. The system was then filled with ^16^O_2_ or ^18^O_2_ and equilibrated for 5 minutes. The setup was sealed, and the sample was incubated at room temperature for 3 hours. After 3 hours, the gas-tight setup was returned to the anaerobic chamber, and the sample was taken out of the device. Then, 200 µL of 2% HCOOH was added to stop the reaction. The sample was subsequently analysed following the same procedure previously used for inhibition assays.

For experiments in ^18^O-water, KDM4E was buffer exchanged into HEPES buffer (50 mM in ^18^O-water (>98 atom % ^18^O), pH 7.5) using Zeba™ Spin Desalting Columns (ThermoFisher, UK). All cofactor stock solutions were prepared in ^18^O-water, then diluted to the final working concentration using HEPES buffer (50 mM in ^18^O-water (>98 atom % ^18^O), pH 7.5). Assay conditions were as above.

### LC–MS/MS with H2a(1–20)

The products of KDM4E (or no enzyme) incubation with H2a(1–20) (conditions in Supplementary Table 6) were analysed using a NanoAcquity-UPLC system (Waters) connected to an Orbitrap Elite MS machine (Thermo Fischer Scientific) with an EASY-Spray nano-electrospray ion source (Thermo Fischer Scientific). Peptides were trapped on an in-house packed guard column (75 μm i.d. × 20 mm, Acclaim PepMap C18, 3μm, 100 Å) using Solvent A (0.1% (v/v) HCOOH in water) at a pressure of 140 bar, then separated using an EASY-spray Acclaim PepMap® analytical column (75 μm i.d. × 15 mm, RSLC C18, 3 μm, 100 Å) employing a linear gradient (length: 100 minutes, 3% to 60% (v/v) Solvent B (0.1% (v/v) HCOOH in acetonitrile); flow rate: 300 nL min^−1^).

Data were collected in the data-dependent mode using electron-transfer dissociation (ETD) fragmentation. Full-scan MS spectra (scan range 350–1500 m/z, resolution 120,000, AGC target 1e6, maximum injection time 250 ms) and subsequent ETD MS/MS spectra (AGC target 5e4, maximum injection time 100 ms) of the ten most intense peaks were acquired. ETD fragmentation was performed at 120 ms activation time collision energy; the signal intensity threshold was maintained at 500 counts. Data analysis used PEAKS 8.5 (Bioinformatics Solutions Inc). The raw MS file was searched against the respective protein sequence. Carbamidomethylation (cysteine), oxidation (methionine), carbamylation (*N*-terminus), deamination (asparagine, glutamine), arginine and lysine methylation, and the custom hydroxylation were set as variable modifications. The precursor mass tolerance was 15 ppm. Fragment mass tolerances were 0.8 Da. The spectra of peptides demonstrating any modifications were manually checked, validated, or rejected.

### Assays with recombinant histone H2a and KDM4E

Recombinant human histone H2a(1–129) (New England Biolabs) was buffer exchanged from 1 mM EDTA, 30 mM NaCl, 20 mM sodium phosphate buffer (pH 7.5) to 50 mM HEPES (pH 7.5) using a Micro Bio-Spin chromatography column P6 (BIO-RAD), according to the manufacturer’s instructions. The resultant concentration was 0.6 mg mL^−1^ (using a Nanodrop spectrophotometer ND-1000, Thermo Scientific). The following mixture was incubated at room temperature (16 hours) in duplicate: H2a(1–129) (35 µM), 14 µM KDM4E (or no enzyme), 100 µM 2OG, 100 µM sodium L-ascorbate, 10 (NH_4_)_2_Fe(SO_4_)_2_, and 50 mM HEPES (pH 7.5). 1:1 (v/v) 1% HCOOH in ddH_2_O was added to quench the reaction.

MALDI–TOF MS analyses of recombinant histone H2a (treated/untreated with KDM4E). The sample (2 µL) was mixed with 2 µL of 2% (v/v) aqueous trifluoroacetic acid (CF_3_CO_2_H) solution. MALDI matrix, was prepared by adding 7.6 mg of 2,5-dihydroxyacetophenone (2,5-DHAP) in 375 µL ethanol to 125 µL of an aqueous solution of diammonium hydrogen citrate (18 mg mL^−1^). The mixture (2 µL) was added to the acidified sample solution; the resultant material was mixed and crystallisation initiated by pipetting. The crystalline suspension (0.5 µL) was pipetted onto the target ground steel MALDI plate (Bruker) and allowed to dry. The samples were analysed using MALDI–TOF MS in linear mode using the default manufacturer settings for TOF/TOF (Bruker Autoflex). FlexControl v3.4 was used for data collection and FlexAnalysis v3.4 for data analysis.

MALDI Top-Down Sequencing (TDS) of recombinant histone H2a (un/treated). KDM4E treated or untreated histone H2a(1–129) samples (1 µL) were added to 1 µL of a 50 mg mL^−1^ super-dihydroxybenzoic acid (DHB) (i.e. 9:1 (w/w) mixture of 2,5-DHB and 2-hydroxy-5-methoxybenzoic acid) in TA50 (50:50 acetonitrile: 0.1% (v/v) CF_3_CO_2_H in ddH_2_O)]. This mixture (0.5 µL) was spotted onto a ground steel MALDI plate and allowed to dry. Samples were subjected to MALDI TDS using ISD (top-down sequencing of intact proteins) with the manufacturer’s settings for TOF/TOF (Bruker Autoflex). FlexControl v3.4 was used for data collection and FlexAnalysis v3.4 and Biotools for data analysis.

### LC–MS/MS analysis of enzyme assays with calf thymus histones and KDM4E

KDM4E-treated and untreated samples analysed by LC–MS (see above) were analysed by LC–MS/MS. A solution of 2 μL of 85 mM dithiothreitol (DTT) in 50 mM ammonium bicarbonate (AmBic) was added to ∼15 μg of protein. The mixture was incubated for 40 minutes at 56 °C. Then 7 μL of 55 mM iodoacetamide in 50 mM AmBic was added, and the mixture was incubated in the dark (room temperature, 30 minutes). 3 μL of 85 mM DTT in 50 mM AmBic were then added. Trypsin (Promega) at a 1:50 (w/w) ratio to the total protein amount was added and the mixture incubated (37 °C, 16 hours). A second tryptic digestion (3 hours, 37 °C) with a ratio of 1:100 trypsin to total protein was then carried out. Acetonitrile was then added to a final concentration of 80% (v/v) acetonitrile; HCOOH was then added to a final concentration of 5% (v/v). Samples were then dried using a vacuum centrifuge (Eppendorf Concentrator) and dissolved in 10–20 μL of 0.1% (v/v) HCOOH in ddH_2_O. ZipTip pipette tips (C18, Merck Millipore) were used to purify the samples according to the manufacturer’s instructions. The purified samples were eluted in 20 μL of 60% (v/v) acetonitrile, 40% (v/v) ddH_2_O and 0.1% (v/v) HCOOH.

Samples were analysed using a NanoAcquity-UPLC system (Waters) connected to an Orbitrap Elite mass spectrometer (Thermo Fischer Scientific) with an EASY-Spray nano-electrospray ion source (Thermo Fischer Scientific). The peptides were trapped on a guard column (75 μm i.d. × 20 mm, Acclaim Pepmap100 C18, 3μm, 120 Å) using Solvent A (0.1% (v/v) HCOOH in ddH_2_O) at a pressure of 140 bar, then separated on an EASY-spray Acclaim PepMap® analytical column (75 μm i.d. × 15 mm, RSLC C18, 3 μm, 100 Å) using a 68 minute linear gradient from 3 to 97% (v/v) of solvent B (0.1% (v/v) HCOOH in acetonitrile) (flow rate: 300 nL min^−1^; column temperature: 40 °C). The nESI source was operated at a needle voltage of 1600 V; the ion transfer tube temperature was 275 °C. Data were collected in the data-dependent mode using a CID-based method. Full scan MS spectra (350–1500 m/z, resolution 120,000, AGC target 1e6, maximum injection time 250 ms) and subsequent CID MS/MS spectra (AGC target 5e4, maximum injection time 100 ms) of the 10 most intense peaks were acquired. CID fragmentation was performed at 35% of the normalised collision energy and the signal intensity threshold was kept at 500 counts. Analysis was performed as described above (LC–MS/MS with H2a(1–20) peptide) with the addition of selecting trypsin as the protease with a maximum of three missed cleavages and one non-specified end.

NMR analysis for assignment of H2aR20 hydroxylation regiochemistry. H2a(1–20) (~2.1 mg) was incubated with 50 µM KDM4E, 10 µM (NH_4_)_2_Fe(SO_4_)_2_, 100 µM sodium L-ascorbate, 200 µM 2OG, and 50 mM HEPES pH 7.5 (8 mL final volume) for 16 hours at room temperature; the reaction was quenched by 1:1 (v/v) 1% aqueous HCOOH. The mixture was desalted using two Sep Pak C18 columns (Waters) in tandem, eluting with 4 mL of 40% acetonitrile in ddH_2_O and 0.1% (v/v) HCOOH in ddH_2_O. Samples were frozen, lyophilised, and suspended in 160 µL H_2_O/D_2_O (9:1) for NMR analyses (HSQC, COSY, and TOCSY) using a Bruker AVIII 700 MHz NMR spectrometer equipped with a 5 mm inverse cryoprobe using 3 mm MATCH NMR tubes (Cortecnet). Data were processed using TopSpin 3.2 software (Bruker).

#### Statistics and reproducibility

Where relevant, the number of independent assays and biological replicates are indicated in the figure legends. In most cases, error bars represent standard deviation (SD) as a measure of data dispersion from the mean with the exception of Michaelis–Menten kinetic plots where standard error mean (SEM) was used.

### Reporting summary

Further information on research design is available in the Nature Portfolio Reporting Summary linked to this article.

## Supplementary information

Supplementary Information

Description of Additional Supplementary Files

Supplementary Data

Reporting Summary

## Data Availability

All data generated or analysed during this study are included in this published article (and in Supplementary Information files). Source data can be obtained from supplementary source data file and in Oxford University Research Archive (ORA)^68^. All other data are available from the corresponding authors (or other sources, as applicable) on reasonable request.
