# Supplementary material for: JmjC catalysed histone H2a N-methyl arginine demethylation and C4-arginine hydroxylation reveals importance of sequence-reactivity relationships
Source: Commun Biol. 2024 Nov 27;7:1583. doi: 10.1038/s42003-024-07183-5 (PMC11603075; doi:10.1038/s42003-024-07183-5)
Supplement: Supplementary file 1 — Supplementary Information [file 42003_2024_7183_MOESM1_ESM.pdf]

Supplementary Information

JmjC catalysed histone H2a *N*-methyl arginine demethylation and C4-arginine hydroxylation reveals importance of sequence-reactivity relationships

Joanna Bonnici[1,2], Razanne Oueini[1], Eidarus Salah[1], Catrine Johansson[3], Elisabete Pires[1], Martine Abboud[1], Robert S. Dawber[2], Anthony Tumber[1], Patrick Rabe[1], Hilal Saraç[1,2], Christopher J. Schofield[1*], and Akane Kawamura[1,2*]

[1] Chemistry Research Laboratory, Department of Chemistry and the Ineos Oxford Institute for Antimicrobial Research, University of Oxford, Oxford OX1 3TA, United Kingdom.

[2] Chemistry - School of Natural and Environmental Sciences, Newcastle University, Newcastle Upon Tyne, NE1 7RU, United Kingdom.

[3] Botnar Research Centre, NIHR Oxford Biomedical Research Unit, University of Oxford, Oxford, OX3 7LD, United Kingdom.

*Correspondence:

christopher.schofield@chem.ox.ac.uk; akane.kawamura@newcastle.ac.uk

Keywords: JmjC 2-oxoglutarate / α-ketoglutarate oxygenase, histone *N*-methyl arginine / lysine demethylase, arginine hydroxylation, epigenetics, regulation of transcription, histone post-translational modification, histone H2a.

Table of Contents

Supplementary Figure 1 Initial Screen of H2a fragments with KDM4A 1

Supplementary Figure 2 Histone H2a peptide fragments are substrates of KDM4s and KDM5s. 2

Supplementary Figure 3 KDM4E catalysed H2a(1–20) hydroxylation and H3(1–21)R2me2a demethylation. 3

Supplementary Figure 4 Activity of KDM5C (black circles) with H2a(1–20)R3me2a is greater than that of KDM4E (white circles). 4

Supplementary Figure 5 H2aR3me2a is a substrate of full length KDM4A. 5

Supplementary Figure 6 Conversion of H2a peptides with increased concentrations of KDM4D. 6

Supplementary Figure 7 Hydroxylation is 2OG-dependent and inhibited by broad spectrum 2OG oxygenase inhibitors. 7

Supplementary Figure 8 Hydroxylation is O_2-dependent. 8

Supplementary Figure 9 KDM4E-catalysed hydroxylation of Arg 20 of histone H2a peptide fragment. 10

Supplementary Figure 10 KDM, RDM and hydroxylation (+16 Da) activities of KDM4E are sequence and context dependent 13

Supplementary Figure 11 Evidence arginine hydroxylation by KDM4E occurs at C-4 of H2a R20. 14

Supplementary Figure 12 Hydroxylation and demethylation of H2a(1–20)R20meX by KDM4E 17

Supplementary Figure 13 Reactivity of KDM4D with the H2a peptide panel 18

Supplementary Figure 14 Evidence KDM4E catalyses hydroxylation of full-length histone H2a at R20. 20

Supplementary Figure 15 Evidence KDM4E catalyses hydroxylation of full-length calf thymus histone H2a at R20. 21

Supplementary Figure 16 An artefact is formed when the product of H2a(1–20) and KDM4E is analysed using α-cyano-4-hydroxycinnamic acid (HCCA) and MALDI–TOF MS. 22

Supplementary Figure 17 Comparison of specific hydroxylation activities of KDM4E with H2a(1–20) when analysed by MALDI-TOF MS or LC–MS. 23

Supplementary Table 1 Reported post-translational modifications (PTMs) on the N-terminus of histone H2a. 24

Supplementary Table 2 Comparison of sequences surrounding lysine and arginine demethylation substrates of KDM4E. 25

Supplementary Table 3 Comparison of HAR domain sequences of histone H2a across species. 26

Supplementary Table 4 Summary of constructs, expression systems used and characterisation of purified enzymes. 28

Supplementary Table 5 Peptides used. 29

Supplementary Table 6 Summary of MS assay conditions for substrate screening. 30

Supplementary References 31

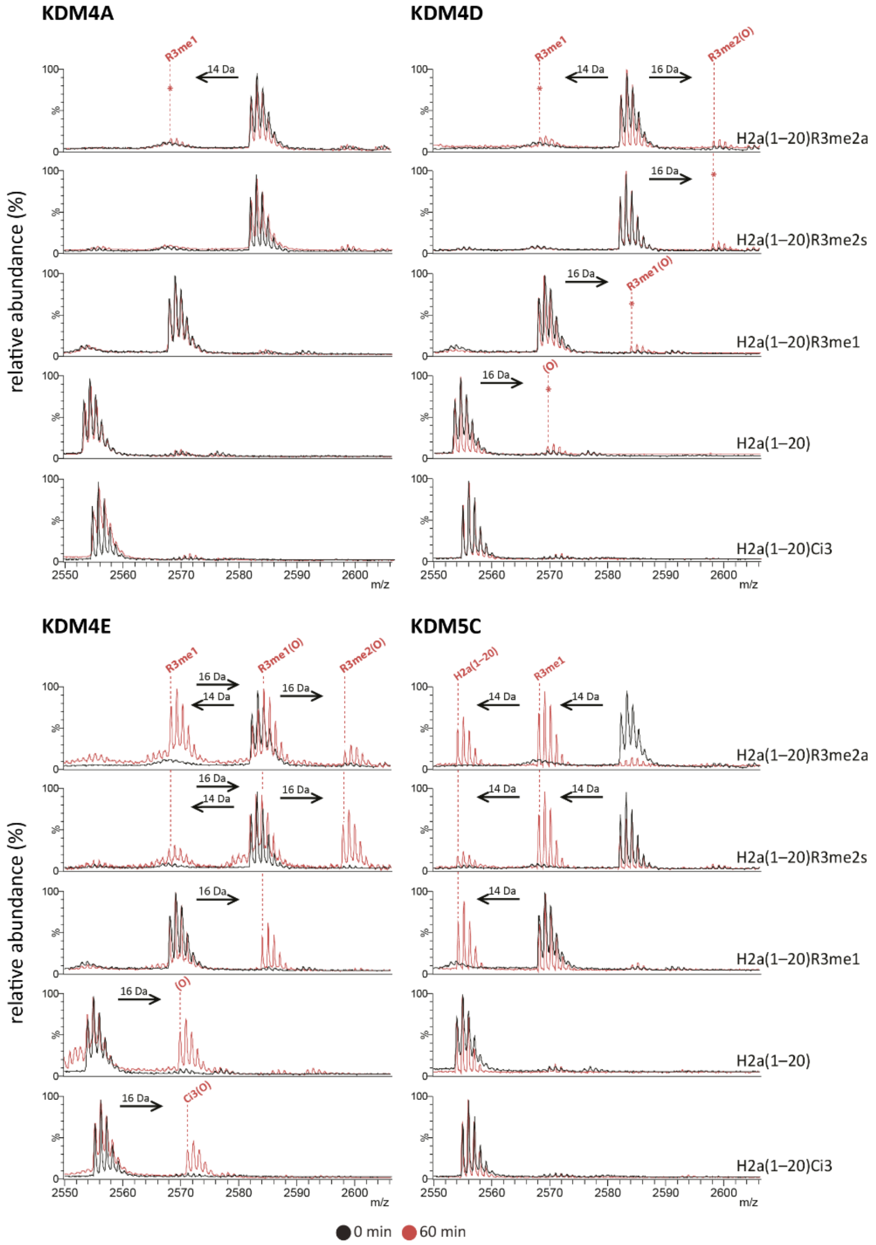

Supplementary Figure 1 Initial Screen of H2a fragments with KDM4A, KDM4D, KDM4E, and KDM5C. Representative MALDI–TOF MS spectra following 60-minute incubations of **(from left to right)** KDM4A, KDM4D, KDM4E, and KDM5C with H2a(1–20)R3X peptides from the AltaBioscience peptide library (Set 5) **(from top to bottom)**: H2a(1–20)R3me2a, H2a(1–20)R3me2s, H2a(1–20)R3me1, H2a(1–20), and H2a(1–20)Ci3. −14 Da mass shifts, corresponding to demethylation, were observed for KDM4A, KDM4D (potentially), KDM4E, and KDM5C with H2a(1–20)R3me2a; KDM4E and KDM5C with H2a(1–20)R3me2s; and KDM5C with H2a(1–20)R3me1. +16 Da mass shifts corresponding to hydroxylation, were observed with KDM4D (potentially) and KDM4E with H2a(1–20)R3me2a, H2a(1–20)R3me2s, H2a(1–20)R3me1, H2a(1–20), and H2a(1–20)Ci3 (only with KDM4E). No mass shifts were observed for KDM4D and KDM5C with H2a(1–20)Ci3 and KDM5C with H2a(1–20). No mass shifts were observed for KDM4A with any of the peptides except H2a(1–20)R3me2a. * Low-level peaks indicating ~10% demethylation. Experiments were repeated with the same results. For assay conditions see **Supplementary Table 6** (n = 2, independent assays). Peptides were synthesised linked with C-terminal N^{ε}-(D-biotin)-L-lysine-amide group (see **Supplementary Table 5**). Charge state of labelled ions: $[MH]^{+}$.

a)

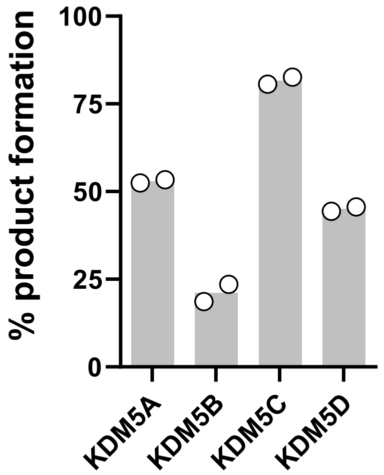

b)

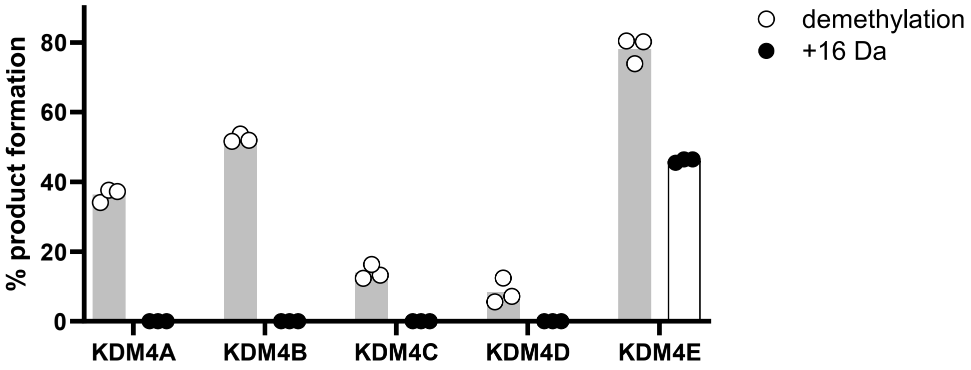

Supplementary Figure 2 Histone H2a peptide fragments are substrates of KDM4s and KDM5s. See **Figure 2** for representative corresponding MALDI–TOF MS traces. Bar graphs comparing % product formation (+16 Da and demethylation) of H2a(1–20)R3me2a by **(a)** KDM5A–D (after 90 minutes) and **(b)** KDM4A–E (after 60 minutes), analysed by MALDI–TOF MS. n = **(a)** 2, **(b)** 3 (independent assays). See **Supplementary Table 6** for assay conditions. See **Figure S13A**[1] for a positive control.

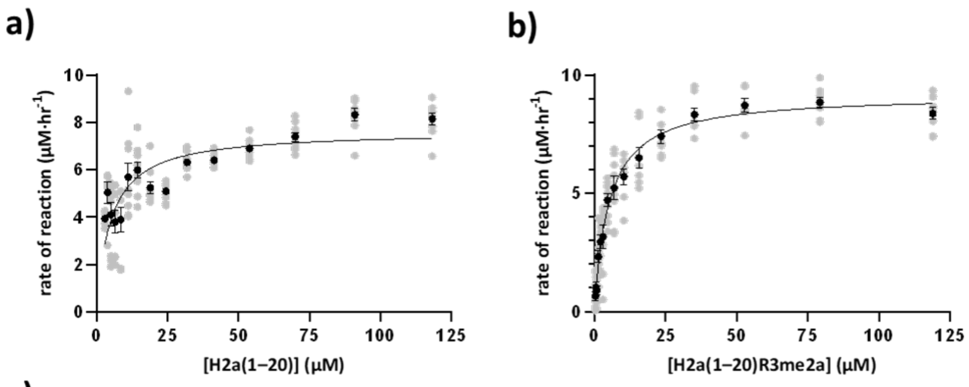

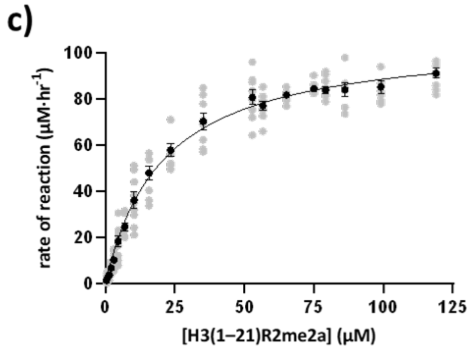

d)

Peptide	K_M (µM) ± SEM	k_{cat} (hr^{-1}) ± SEM	k_{cat}/K_M
H2a(1–20)	4.96 ± 0.66	3.82 ± 0.11	0.77
H2a(1–20)R3me2a	5.18 ± 0.41	4.59 ± 0.10	0.89
H3(1–21)R2me2a	20.69 ± 1.25	53.44 ± 0.97	2.58

Supplementary Figure 3 KDM4E catalysed H2a(1–20) hydroxylation and H3(1–21)R2me2a demethylation. Michaelis–Menten plots monitoring: **(a)** hydroxylation of H2a(1–20) measured using LC–MS (R^2 value obtained was 0.5 indicating poor goodness of fit, likely due to limited data at low substrate concentrations due to signal:noise limitations), **(b)** demethylation of H2a(1–20)R3me2a, and **(c)** demethylation of H3(1–21)R2me2a measured using the FDH-coupled demethylation assay. **(d)** Kinetic parameters obtained in **(a–c)**. Error bars: SEM, n = 3 assay repeats; each assay replicate has n = 3 independent assays. Conditions: LC–MS assay: 2 µM KDM4E, varied concentrations of peptide, 10 µM $(NH_4)_2Fe(SO_4)_2$, 100 µM sodium L-ascorbate, 200 µM 2OG, and 50 mM HEPES pH 7.5, FDH assay: 2 µM KDM4E, varied concentrations of peptide, 10 µM $(NH_4)_2Fe(SO_4)_2$, 100 µM sodium L-ascorbate, 200 µM 2OG, 50 mM HEPES pH 7.5, 0.01% Tween, 500 µM β-NAD, 1 µM FDH.

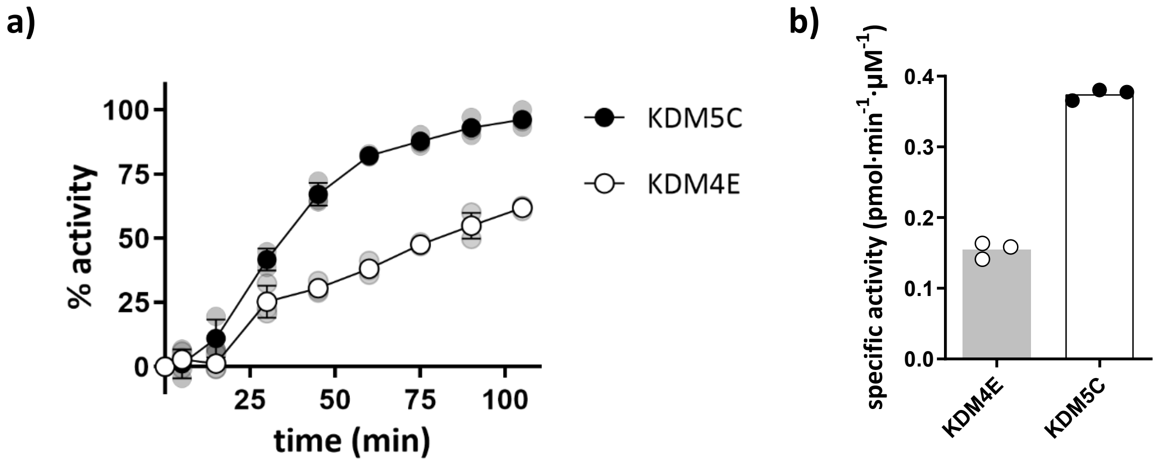

Supplementary Figure 4 Activity of KDM5C (black circles) with H2a(1–20)R3me2a is greater than that of KDM4E (white circles). (a) A MALDI–TOF MS time course graph of product formation (%) of KDM4E (−14 Da/+16 Da) and KDM5C (−14 Da) reaction with H2a(1–20)R3me2a versus time (minutes). ~60% substrate depletion was reached after 105 minutes with KDM4E; ~95% of H2a(1–20)R3me2a was demethylated after 105 minutes with KDM5C. **(b)** Comparison of specific activities of KDM4E with H2a(1–20)R3me2a (0.15 pmol·min^{-1}·µM^{-1}; SD: 0.010) and KDM5C with H2a(1–20)R3me2a (0.37 pmol·min^{-1}·µM^{-1}; SD 0.006). Error bars; +/- SD; n = 3 (independent assays). See **Supplementary Table 6** for assay conditions.

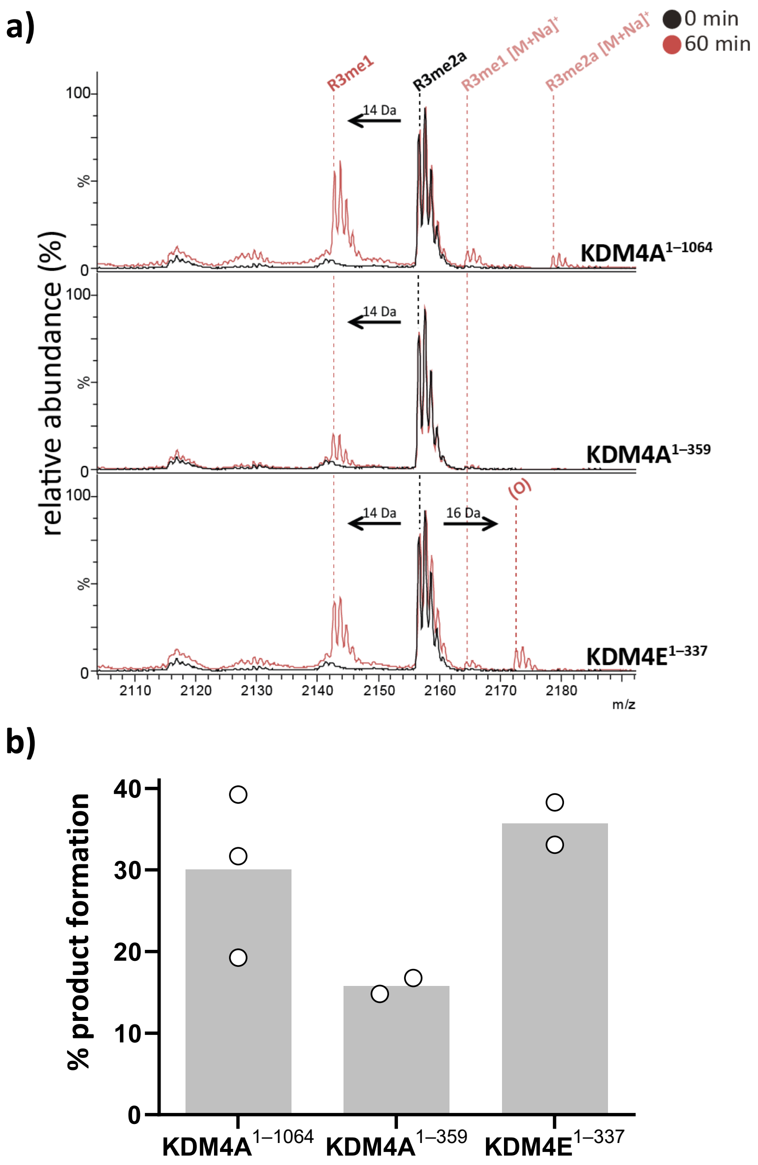

Supplementary Figure 5 H2aR3me2a is a substrate of full length KDM4A. (a) Representative MALDI–TOF MS (60-minute incubation) **(from top to bottom)** KDM4A$^{1-1064}$, KDM4A$^{1-359}$ and KDM4E$^{1-337}$ with H2a(1–20)R3me2a showing –14 Da mass shifts (demethylation) and a +16Da mass shift in the case of KDM4E. Representative data are shown from n = 3 (independent assays). Charge state of labelled ions: $[MH]^+$. **(b)** Bar graph comparing % product formation of H2a(1–20)R3me2a by KDM4A$^{1-1064}$, KDM4A$^{1-359}$ and KDM4E$^{1-337}$, analysed by MALDI–TOF MS. n = 2/3 (independent assays). Conditions: 1 µM KDM4A$^{1-359}$ and KDM4E$^{1-337}$, 10 µM peptide, 100 µM 2OG, 100 µM sodium L-ascorbate, 10 µM $(NH_4)_2Fe(SO_4)_2$, and 50 mM HEPES (pH 7.5); 1 µM KDM4A$^{1-1064}$, 10 µM peptide 100 µM 2OG, 100 µM sodium L-ascorbate, 50 µM $(NH_4)_2Fe(SO_4)_2$, and 50 mM HEPES (pH 7.5), 1 mM TCEP, 0.02% (v/v) Triton-X. See **Figure S12**[1] for positive control.

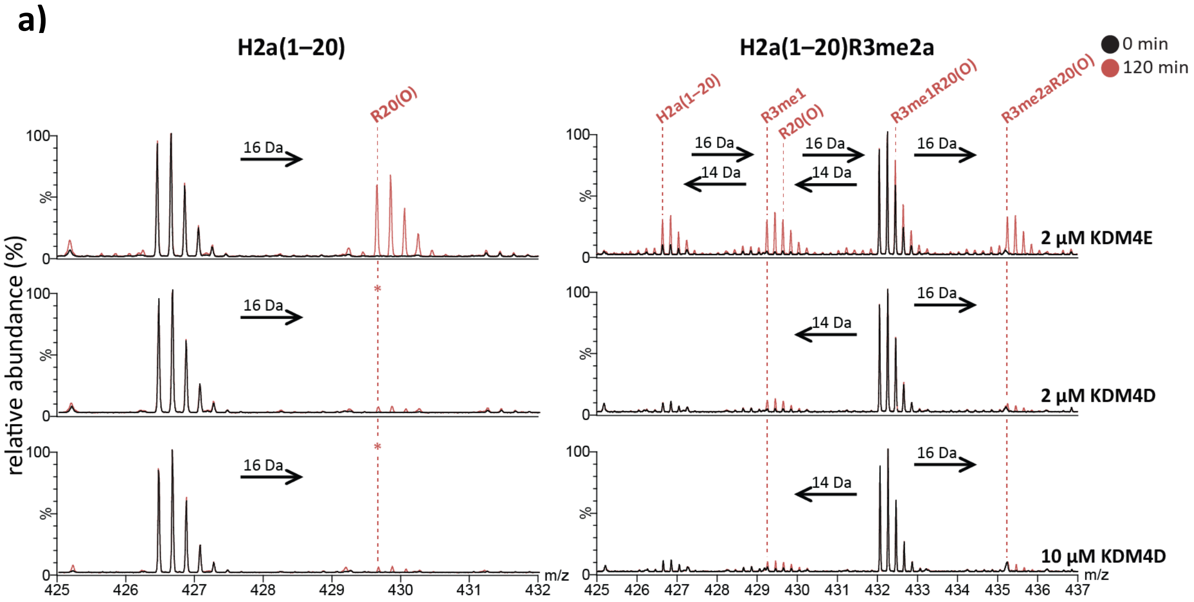

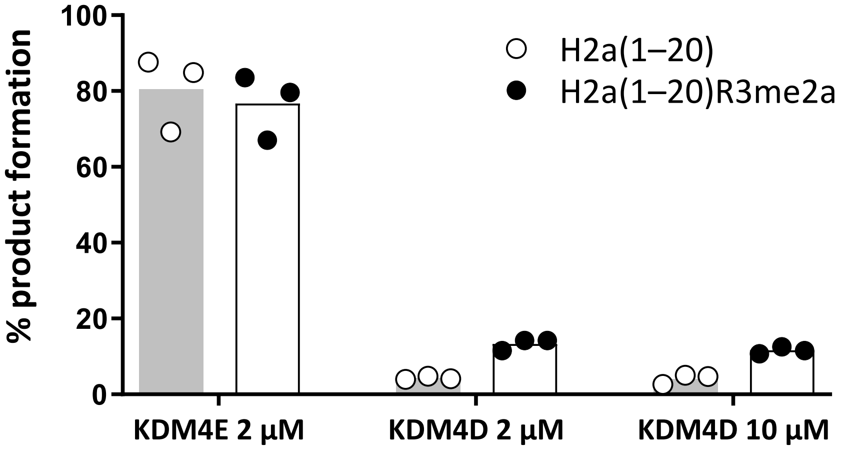

Supplementary Figure 6 Conversion of H2a peptides with increased concentrations of KDM4D. Comparison of combined RDM and apparent hydroxylation activities of KDM4E and KDM4D with H2a(1–20) and H2a(1–20)R3me2a. **(a)** Representative LC–MS spectra showing (120-minute incubation) **(from top to bottom)** KDM4E at 2 µM, KDM4D at 2 µM, and KDM4D at 10 µM with **(from left to right)** H2a(1–20) and H2a(1–20)R3me2a showing multiple −14 Da mass shifts corresponding to demethylation and +16 Da mass shift (hydroxylation). Charge state of labelled ions: $[M + H]^{5+}$. *Low-level peaks indicating ~5% –14 Da/+16 Da. **(b)** Bar graph comparing % product formation by KDM4E and KDM4D of H2a(1–20) and H2a(1–20)R3me2a; n = 3 (independent assays). See **Supplementary Table 6** for assay conditions. See **Figure S14**[1] for a positive control.

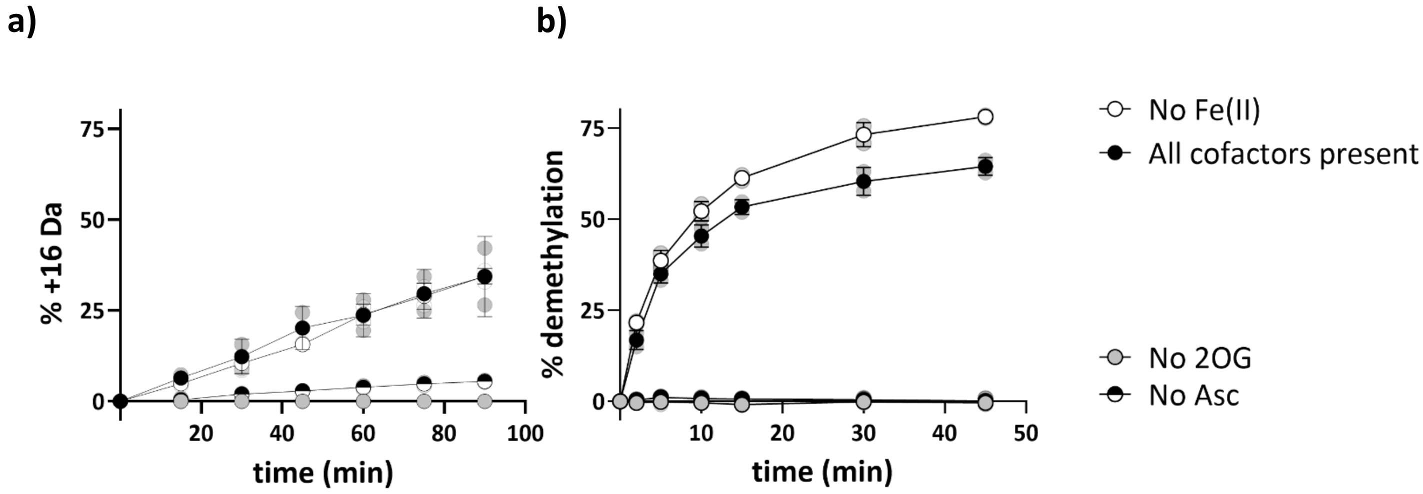

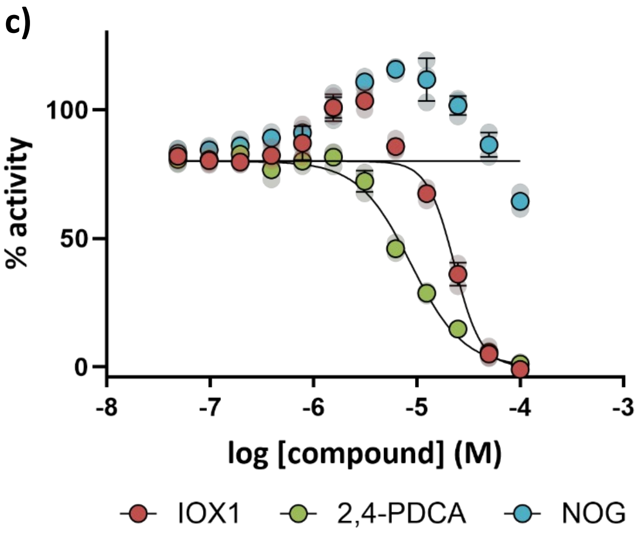

d)

		KDM H3(1–15) K9me2	RDM H3(1–15) R2me2a	ROH H2a(1–20)
Inhibitor		pIC50 (KDM4E)		
IOX1	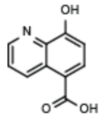	5.65[2]	5.45[2]	4.64
2,4-PDCA	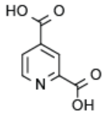	6.75[2]	5.96[2]	5.05
NOG	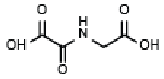	5.06[2]	3.91[2]	<4.00

Supplementary Figure 7 Hydroxylation is 2OG-dependent and inhibited by broad spectrum 2OG oxygenase inhibitors. MALDI–TOF MS time courses measuring **(a)** +16 Da product formation with H2a(1–20) and **(b)** demethylation of H3(1–21)K9me3 by KDM4E. Time courses with all components (i.e. $(NH_4)_2Fe(SO_4)_2$ (Fe(II)), sodium L-ascorbate (Asc) and 2OG), or with one missing component (Fe(II), ascorbate or 2OG) were done. No reaction was observed without 2OG. Error bars: +/- SD; n = 2 (independent assays). Legend for **(a)** and **(b)** can be found combined in **(b)**. Conditions: **(a)** 2 µM KDM4E, 7 µM H2a(1–20), **(b)** 0.6 µM KDM4E, 10 µM H3(1–21)K9me3, 100 µM 2OG, 100 µM sodium L-ascorbate, 10 µM $(NH_4)_2Fe(SO_4)_2$, and 50 mM HEPES (pH 7.5). **(c,d)** Inhibition of arginine hydroxylation (ROH) activity of KDM4E by small molecule 2OG oxygenase inhibitors. **(c)** ROH activity measurements were performed using an LC–MS based assay (Error bars; +/- SD; n = 3 independent assays). Conditions: 10 µM H2a(1-20) incubated with 1 µM KDM4E, 100 µM sodium L-ascorbate, 10 µM $(NH_4)2Fe(SO_4)_2$ and 100 µM 2OG. Reactions were quenched after 90 min. **(d)** pIC_{50} data for ROH activity in comparison with the reported KDM and RDM activity. Assay conditions for KDM and RDM: 5 µM peptide, 0.25 µM (KDM)/1 µM (RDM) enzyme with 10 µM $(NH_4)_2Fe(SO_4)_2$, 100 µM sodium L-ascorbate, 10 µM 2OG. IOX1 – 8-hydroxyquinoline; 2,4-PDCA – 2,4-pyridine dicarboxylic acid; NOG – N-oxalylglycine.[2]

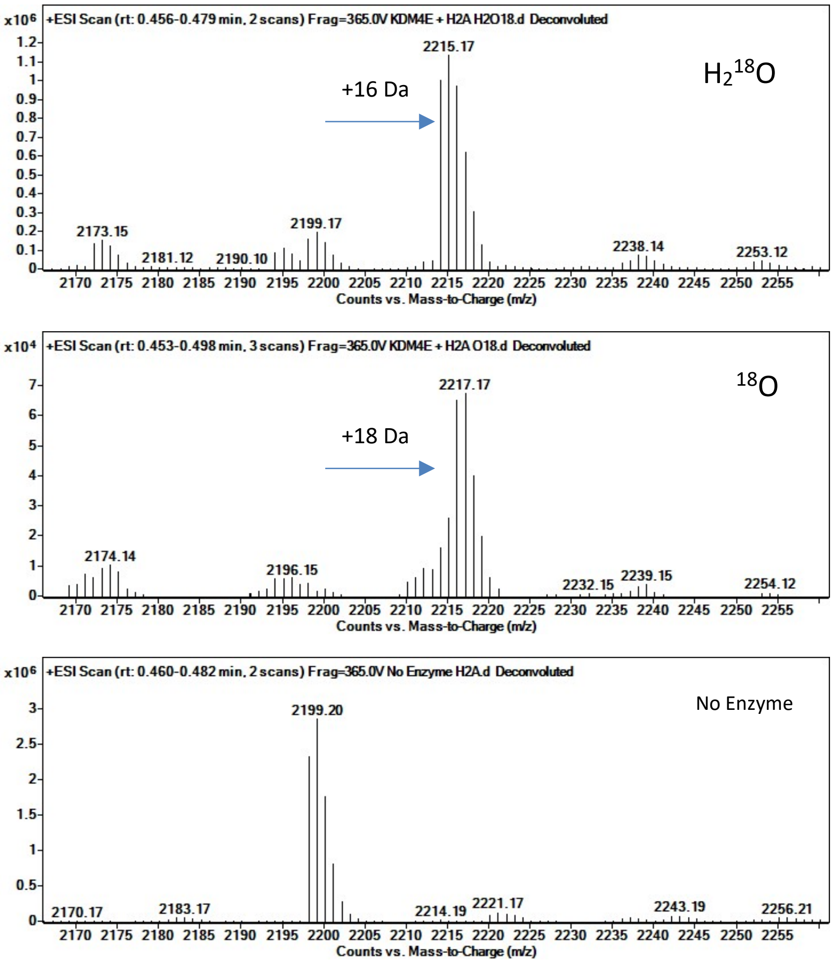

Supplementary Figure 8 Hydroxylation is O_2-dependent. Experiments with $H_2{}^{18}O$ and $^{18}O_2$ with KDM4E and H2a(1–21) analysed using an LC–MS with Q-TOF. MS traces showing 120-minute incubation of KDM4E (10 µM) with H2a(1–21) (20 µM), sodium L-ascorbate (100 µM), 2OG (200 µM), and $(NH_4)_2Fe(SO_4)_2$ (20 µM) in 50 mM HEPES (pH 7.5). **Top:** $H_2{}^{18}O$ experiment showing +16 Da mass shift (relative to no enzyme control, bottom). **Middle:** $^{18}O_2$ experiment showing a +18 Da mass shift (relative to no enzyme control, bottom) confirming incorporation of atmospheric oxygen into the product. **Bottom:** Reaction without added enzyme.

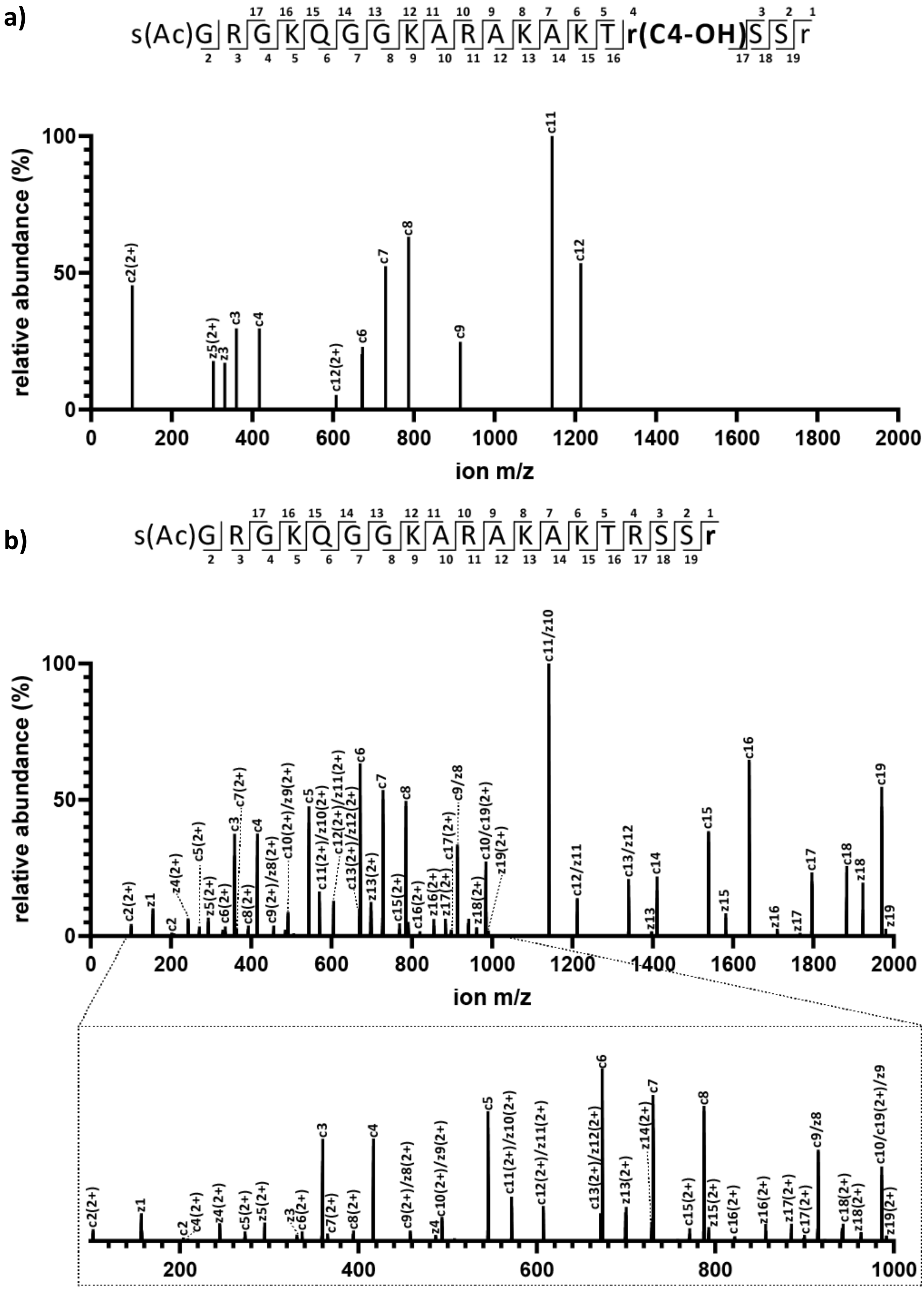
a)
s(Ac)GRGKQGGKARAKAKTr(C4-OH)SSr
relative abundance (%)
ion m/z
c2(2+)
z5(2+)
z3
c3
c4
c12(2+)
c6
c7
c8
c9
c11
c12
b)
s(Ac)GRGKQGGKARAKAKTRSSr
relative abundance (%)
ion m/z
c11/z10
c16
c19
c15
c18
z18
c17
c12/z11
c13/z12
c14
z13
z15
z16
z17
z19
c6
c7
c8
c5
c3
c4
c9/z8
c10/c19(2+)
z19(2+)
c17(2+)
z17(2+)
z16(2+)
c16(2+)
c15(2+)
z13(2+)
c13(2+)/z12(2+)
c12(2+)/z11(2+)
c11(2+)/z10(2+)
c10(2+)/z9(2+)
c9(2+)/z8(2+)
c8(2+)
c7(2+)
c6(2+)
z5(2+)
c5(2+)
z4(2+)
c2(2+)
z1
c2
c4(2+)
z3
z4
z14(2+)
z15(2+)
c10/c19(2+)/z9
c18(2+)
z18(2+)

c)

#	c	c(2+)	Seq	z	z(2+)	#
1	147.08	74.04	S(Ac = +42.01)			20
2	204.10	102.55	G	1998.15	999.58	19
3	360.20	180.60	R	1941.13	971.07	18
4	417.22	209.11	G	1785.03	893.02	17
5	545.32	273.16	K	1728.01	864.50	16
6	673.38	337.16	Q	1599.91	800.46	15
7	730.39	365.70	G	1471.86	736.43	14
8	787.42	394.73	G	1414.83	707.92	13
9	915.51	458.26	K	1357.81	679.84	12
10	986.55	493.78	A	1229.72	615.36	11
11	1142.65	571.83	R	1158.68	579.84	10
12	1213.69	607.35	A	1002.58	501.79	9
13	1341.79	671.39	K	931.54	466.27	8
14	1412.83	706.91	A	803.45	402.22	7
15	1540.91	771.46	K	732.41	366.71	6
16	1641.96	821.48	T	604.32	303.17	5
17	1798.06	899.53	R	503.27	252.64	4
18	1885.10	943.05	S	347.17	174.11	3
19	1972.12	986.55	S	260.13	130.57	2
20			R (Am, C4-OH = +15.01)	173.1	87.05	1

d)

#	c	c(2+)	Seq	z	z(2+)	#
1	147.08	74.04	S(Ac = +42.01)			20
2	204.10	102.55	G	1998.15	999.58	19
3	360.20	180.60	R	1941.13	971.07	18
4	417.22	209.11	G	1785.03	893.02	17
5	545.32	273.16	K	1728.01	864.50	16
6	673.38	337.16	Q	1599.91	800.46	15
7	730.39	365.70	G	1471.86	736.43	14
8	787.42	394.73	G	1414.83	707.92	13
9	915.51	458.26	K	1357.81	679.84	12
10	986.55	493.78	A	1229.72	615.36	11
11	1142.65	571.83	R	1158.68	579.84	10
12	1213.69	607.35	A	1002.58	501.79	9
13	1341.79	671.39	K	931.54	466.27	8
14	1412.83	706.91	A	803.45	402.22	7
15	1540.91	770.96	K	732.41	366.71	6
16	1641.96	821.48	T	604.32	303.17	5
17	1814.06	907.53	R(C4-OH = +15.99)	503.27	252.13	4
18	1901.09	951.05	S	331.17	166.09	3
19	1988.12	994.56	S	244.64	122.57	2
20			R (Am = -0.98)	157.01	79.05	1

e)

#	c	c(2+)	Seq	z	z(2+)	#
1	147.08	74.04	S(Ac = +42.01)			20
2	204.10	102.55	G	1982.16	991.58	19
3	360.20	180.60	R	1925.14	963.07	18
4	417.22	209.11	G	1769.04	885.53	17
5	545.32	273.16	K	1712.01	857.02	16
6	673.38	337.16	Q	1583.92	792.97	15
7	730.39	365.70	G	1455.86	728.94	14
8	787.42	394.73	G	1398.84	700.43	13
9	915.51	458.26	K	1341.79	671.41	12
10	986.55	493.78	A	1213.69	607.35	11
11	1142.65	571.83	R	1142.65	571.84	10
12	1213.69	607.35	A	986.55	493.78	9
13	1341.79	671.39	K	915.51	458.27	8
14	1412.83	706.91	A	787.42	394.73	7
15	1540.91	770.96	K	716.42	359.22	6
16	1641.96	821.48	T	588.32	295.17	5
17	1798.06	900.04	R	487.27	244.64	4
18	1885.11	942.57	S	331.17	166.09	3
19	1972.12	986.55	S	244.64	122.57	2
20			R (Am = -0.98)	157.01	79.05	1

Supplementary Figure 9 KDM4E-catalysed hydroxylation of Arg 20 of histone H2a peptide fragment. LC–MS/MS fragment analysis of H2a(1–20) treated with KDM4E compared to untreated H2a(1–20). The reaction products of KDM4E and H2a(1–20) (+ KDM4E) and a no enzyme control (– KDM4E) H2a(1–20) were analysed using a Hybrid Ion Trap-Orbitrap machine and PEAKS software. No hydroxylation was detected without KDM4E **(b).** With KDM4E two potential hydroxylation sites, R17 (−10logP score: 20.50) **(a)** and R20 (−10logP score: 102.28) (**Figure 3a**) were detected. Complete ion coverage for R20 and a −10logP of 102.28 indicate R20 is likely the hydroxylation site. **(c)** Ion table corresponding to the LC–MS/MS fragment analysis in **Figure 3a** showing fragments indicating potential hydroxylation site at R20**. (d)** Ion table corresponding to LC–MS/MS fragment analysis in **(a)** showing fragments detected for potential R17 hydroxylation. **(d)** Ion table corresponding to LC–MS/MS fragment analysis of the untreated sample indicating a lack of hydroxylation. Greyed out cells: ions not detected.

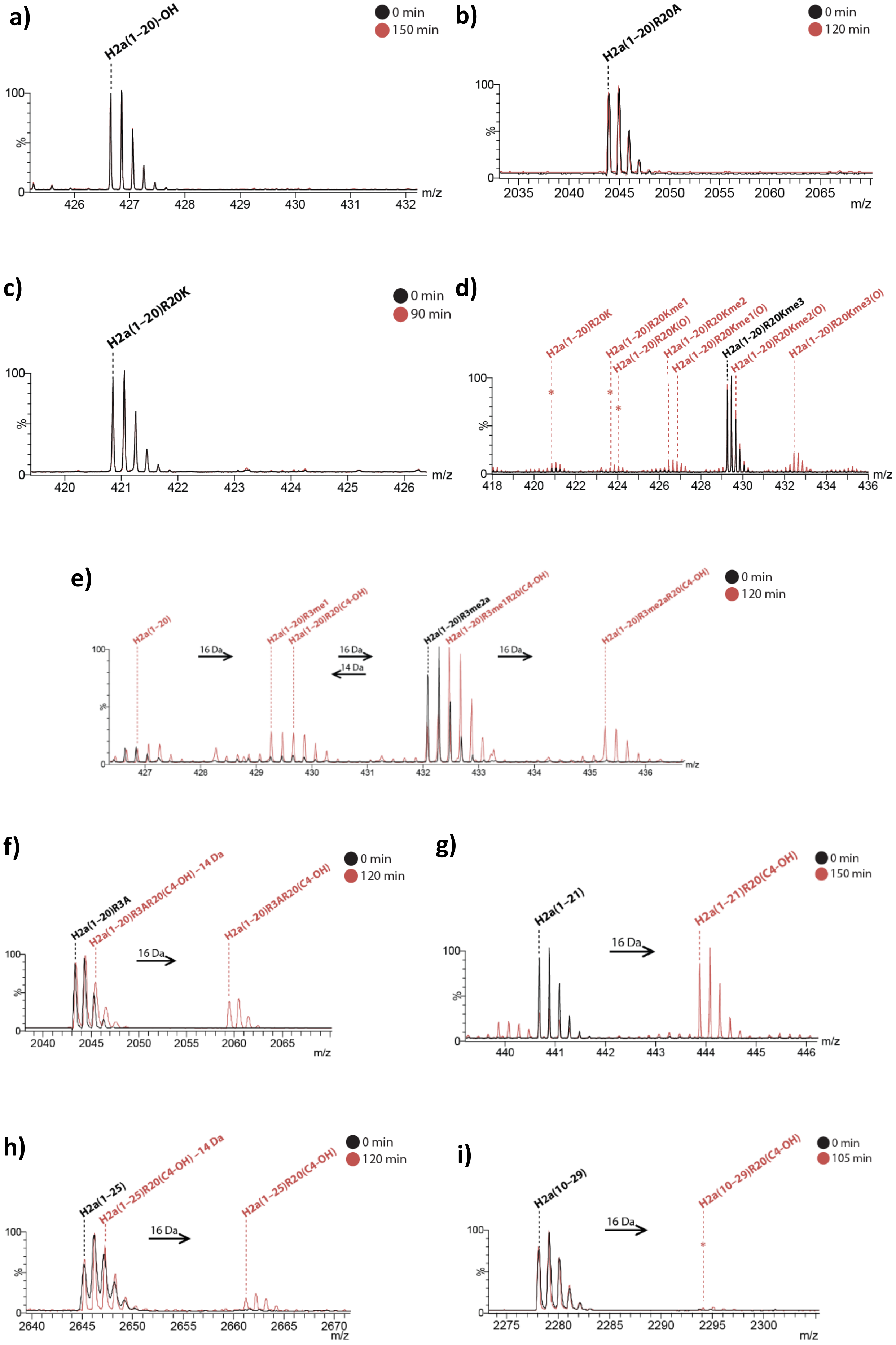
a)
H2a(1–20)-OH
0 min
150 min
100
%
0
426
427
428
429
430
431
432
m/z
b)
H2a(1–20)R20A
0 min
120 min
2035
2040
2045
2050
2055
2060
2065
c)
H2a(1–20)R20K
0 min
90 min
420
421
422
423
424
425
426
d)
H2a(1–20)R20K
H2a(1–20)R20Kme1
H2a(1–20)R20K(O)
H2a(1–20)R20Kme2
H2a(1–20)R20Kme1(O)
H2a(1–20)R20Kme3
H2a(1–20)R20Kme2(O)
H2a(1–20)R20Kme3(O)
0 min
90 min
418
420
422
424
426
428
430
432
434
436
e)
H2a(1–20)
16 Da
H2a(1–20)R3me1
H2a(1–20)R20(C4-OH)
14 Da
H2a(1–20)R3me2a
H2a(1–20)R3me1R20(C4-OH)
H2a(1–20)R3me2aR20(C4-OH)
0 min
120 min
f)
H2a(1–20)R3A
H2a(1–20)R3AR20(C4-OH) –14 Da
H2a(1–20)R3AR20(C4-OH)
0 min
120 min
g)
H2a(1–21)
H2a(1–21)R20(C4-OH)
0 min
150 min
440
441
442
443
444
445
446
h)
H2a(1–25)
H2a(1–25)R20(C4-OH) –14 Da
H2a(1–25)R20(C4-OH)
0 min
120 min
2640
2645
2650
2655
2660
2665
2670
i)
H2a(10–29)
H2a(10–29)R20(C4-OH)
0 min
105 min
2275
2280
2285
2290
2295
2300

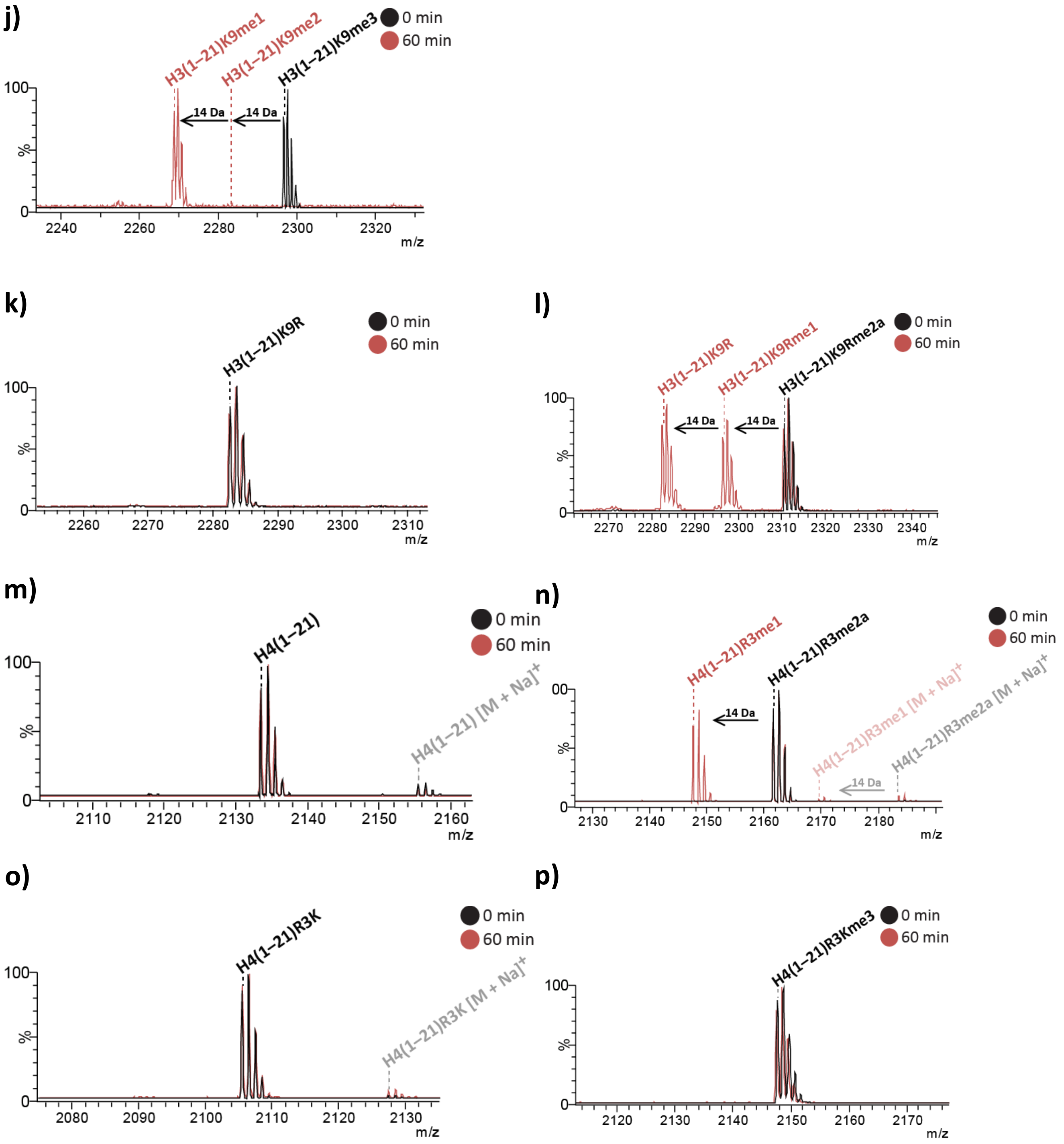
j)
H3(1–21)K9me1
H3(1–21)K9me2
H3(1–21)K9me3
0 min
60 min
14 Da
14 Da
100
%
0
2240
2260
2280
2300
2320
m/z
k)
H3(1–21)K9R
0 min
60 min
100
%
0
2260
2270
2280
2290
2300
2310
m/z
l)
H3(1–21)K9R
H3(1–21)K9Rme1
H3(1–21)K9Rme2a
0 min
60 min
14 Da
14 Da
100
%
0
2270
2280
2290
2300
2310
2320
2330
2340
m/z
m)
H4(1–21)
0 min
60 min
H4(1–21) [M + Na]+
100
%
0
2110
2120
2130
2140
2150
2160
m/z
n)
H4(1–21)R3me1
H4(1–21)R3me2a
0 min
60 min
H4(1–21)R3me1 [M + Na]+
H4(1–21)R3me2a [M + Na]+
14 Da
14 Da
00
%
0
2130
2140
2150
2160
2170
2180
m/z
o)
H4(1–21)R3K
0 min
60 min
H4(1–21)R3K [M + Na]+
100
%
0
2080
2090
2100
2110
2120
2130
m/z
p)
H4(1–21)R3Kme3
0 min
60 min
100
%
0
2120
2130
2140
2150
2160
2170
m/z

Supplementary Figure 10 KDM, RDM and hydroxylation (+16 Da) activities of KDM4E are sequence and context dependent. Representative MALDI–TOF or LC–MS spectra for incubation of KDM4E with: **(a)** H2a(1–20) with a C-terminal carboxylic acid (indicated by -OH), **(b)** H2a(1–20)R20A, and **(c)** H2a(1–20)R20K showing no mass shifts; **(d)** H2a(1–20)R20Kme3, and **(e)** H2a(1–20)R3me2a showing multiple −14 Da mass shifts (demethylation) and a +16 Da mass shift (hydroxylation); **(f)** H2a(1–20)R3A, **(g)** H2a(1–21), **(h)** H2a(1–25) and **(i)** H2a(10–29) showing a +16 Da mass shift; **(j)** H3(1–21)K9me3 showing multiple −14 Da mass shifts; **(k)** H3(1–21)K9R showing no mass shifts; **(l)** H3(1–21)K9Rme2a showing multiple −14 Da mass shifts; **(m)** H4(1–21) showing no mass shifts; **(n)** H4(1–21)R3me2a showing a −14 Da mass shift; **(o)** H4(1–21)R3K and **(p)** H4(1–21)R3Kme3 showing no mass shifts; Charge state of labelled ions: $[M + H]^{5+}$ for **(a)**, **(c–e)**, and **(g)**, and $[MH]^{+}$ for **(b)**, **(f)** and **(h–p)**. *Low level MALDI–TOF MS peaks indicating ~10% product formation. n = 3 (independent assays). Y-axis: relative abundance (%). See **Supplementary Table 6** for assay conditions. See **Figure 3b** for specific activities of H2a peptide panel and **Figure 3c** for summary of K/R substitutions of wild type H2a, H3 and H4 sequences. The –14 Da peak indicated in **(f)** and **(h)** is due to a MALDI–TOF MS derived modification – see **Supplementary Figure 16**.

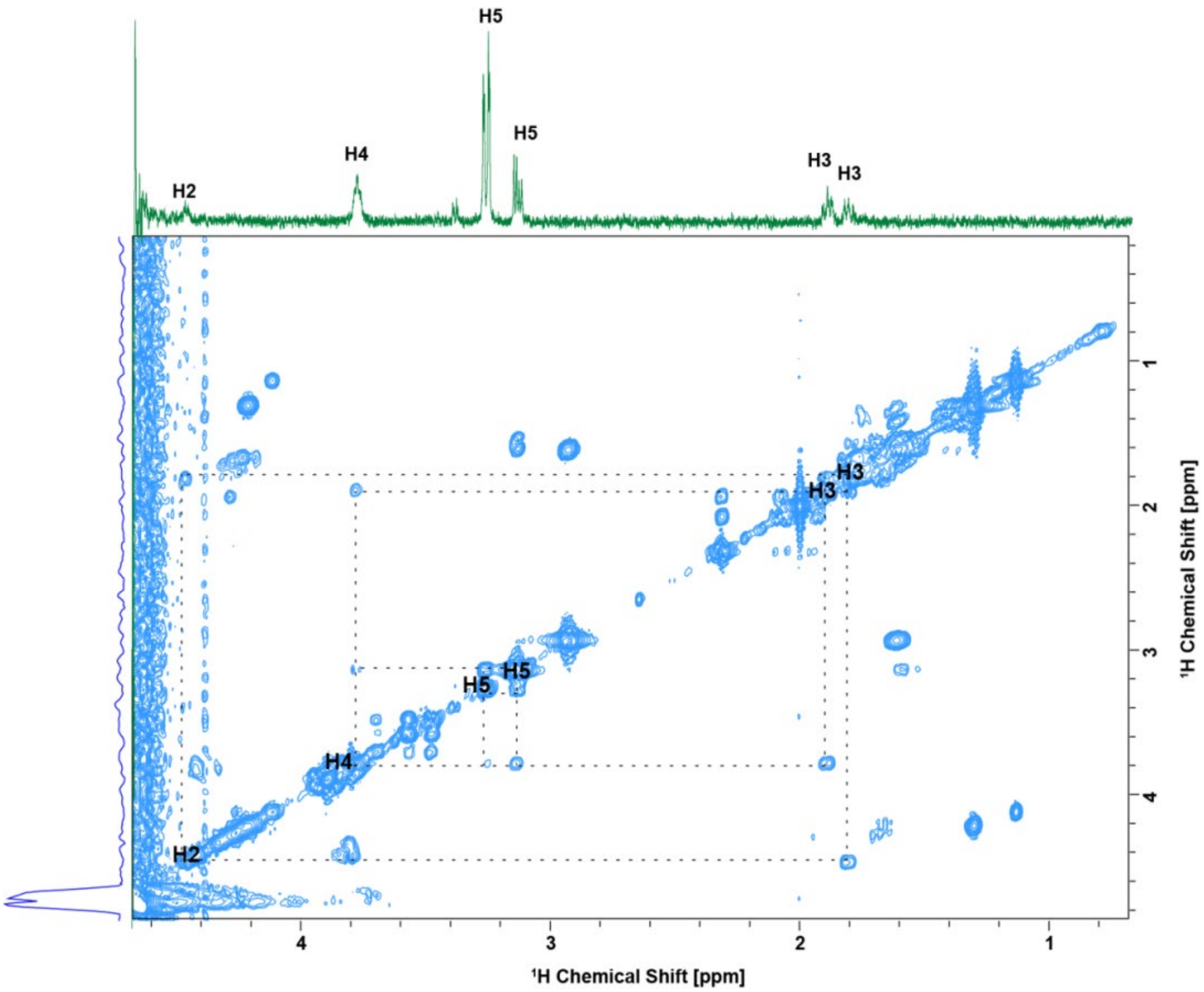

Supplementary Figure 11 Evidence arginine hydroxylation by KDM4E occurs at C-4 of H2a R20. NMR analyses of unmodified and KDM4E-treated H2a(1–20) peptide. ^{1}H-^{1}H COSY spectrum of the hydroxylated H2a peptide, matched with its corresponding 1D TOCSY spectrum, showing correlations of the R20 C-4 C<u>H</u>OH proton. See **Figure 4** for ^{1}H NMR spectra of H2a(1–20) before and after KDM4E treatment and overlay of HSQC spectra of H2a and hydroxylated H2a with the 1D TOCSY spectrum of hydroxylated H2a.

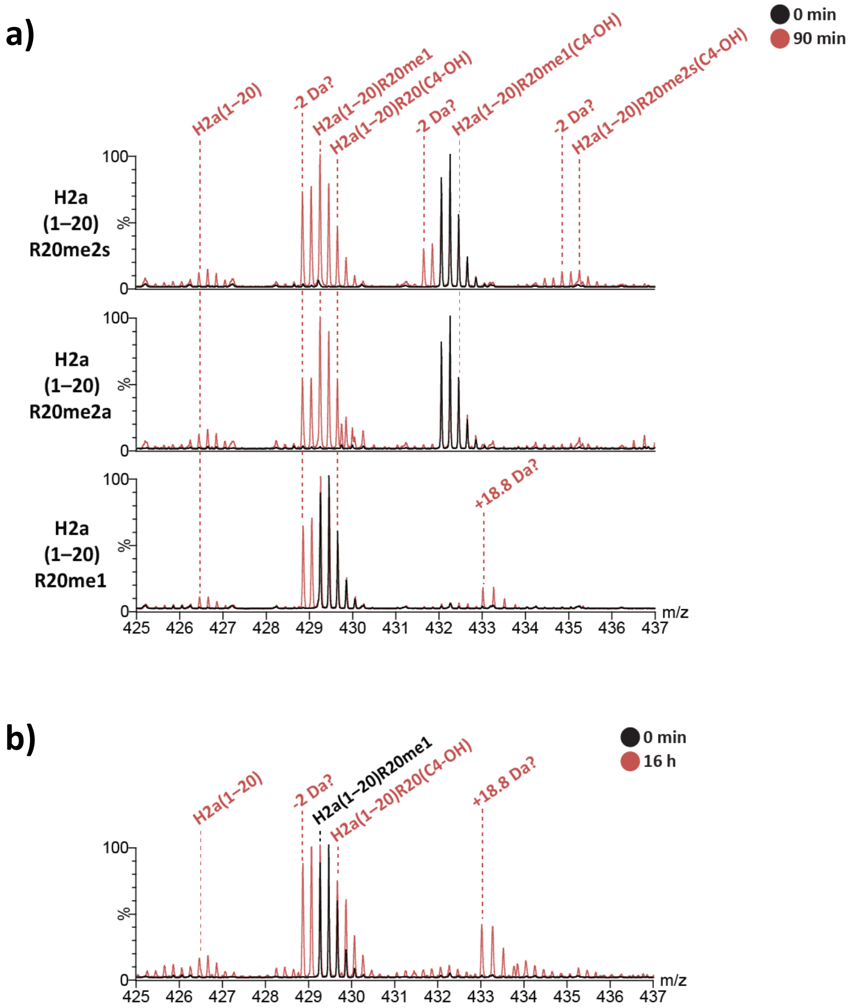

c)

Peptide	expected		observed		mass shift relative to starting material	
	mass (Da)	$[M + H]^{5+}$	mass (Da)	$[M + H]^{5+}$	H2a(1–20)R20me2a/s	H2a(1–20)R20me1
H2a (1–20)R20me2s (C4-OH)	2171.2	435.2	2171.3	435.3	16.0	N/A
H2a (1–20)R20me2s (C4-OH) -2 Da?	N/A	N/A	2169.3	434.9	13.9	N/A
H2a (1–20)R20me1 +18.8 Da?	N/A	N/A	2160.1	433.0	4.8	18.8
H2a (1–20)R20me1(C4-OH)	2157.2	432.4	2157.3	432.5	2.0	16.0
H2a (1–20)R20me2a/s	2155.3	432.1	2155.3	432.1	0.0	N/A
H2a (1–20)R20me2s -2 Da?	N/A	N/A	2153.3	431.7	-2.1	N/A
H2a (1–20)R20(C4-OH)	2143.2	429.6	2143.3	429.7	-12.1	2.0
H2a (1–20)R20me1	2141.2	429.2	2141.3	429.3	-14.1	0.0
H2a (1–20)R20me1 -2 Da?	N/A	N/A	2139.3	428.9	-16.1	-2.0
H2a (1–20)	2127.2	426.4	2127.3	426.5	-28.1	-14.0

d)

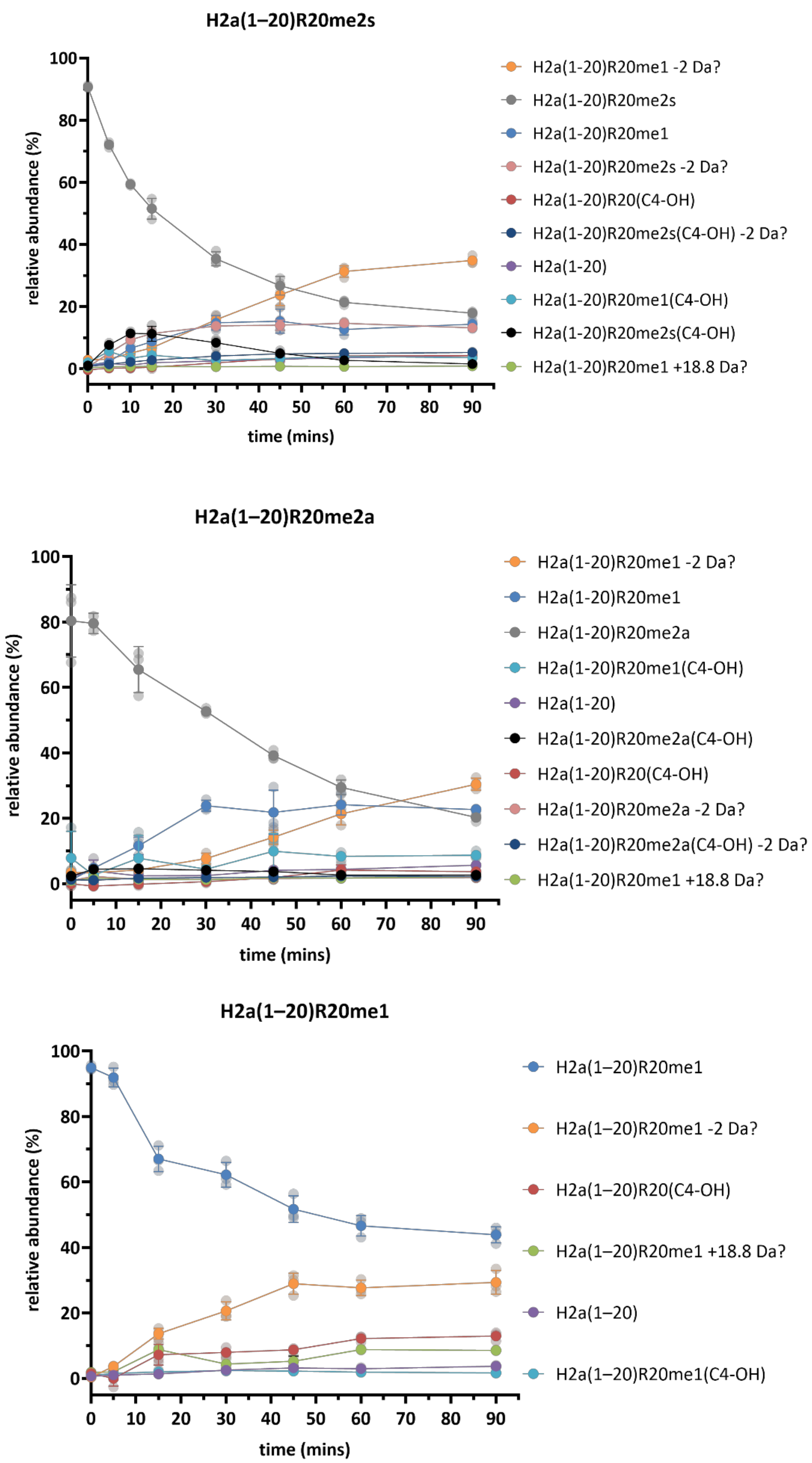
H2a(1–20)R20me2s
relative abundance (%)
time (mins)
H2a(1-20)R20me1 -2 Da?
H2a(1-20)R20me2s
H2a(1-20)R20me1
H2a(1-20)R20me2s -2 Da?
H2a(1-20)R20(C4-OH)
H2a(1-20)R20me2s(C4-OH) -2 Da?
H2a(1-20)
H2a(1-20)R20me1(C4-OH)
H2a(1-20)R20me2s(C4-OH)
H2a(1-20)R20me1 +18.8 Da?
H2a(1–20)R20me2a
relative abundance (%)
time (mins)
H2a(1-20)R20me1 -2 Da?
H2a(1-20)R20me1
H2a(1-20)R20me2a
H2a(1-20)R20me1(C4-OH)
H2a(1-20)
H2a(1-20)R20me2a(C4-OH)
H2a(1-20)R20(C4-OH)
H2a(1-20)R20me2a -2 Da?
H2a(1-20)R20me2a(C4-OH) -2 Da?
H2a(1-20)R20me1 +18.8 Da?
H2a(1–20)R20me1
relative abundance (%)
time (mins)
H2a(1–20)R20me1
H2a(1–20)R20me1 -2 Da?
H2a(1–20)R20(C4-OH)
H2a(1–20)R20me1 +18.8 Da?
H2a(1–20)
H2a(1–20)R20me1(C4-OH)

e)

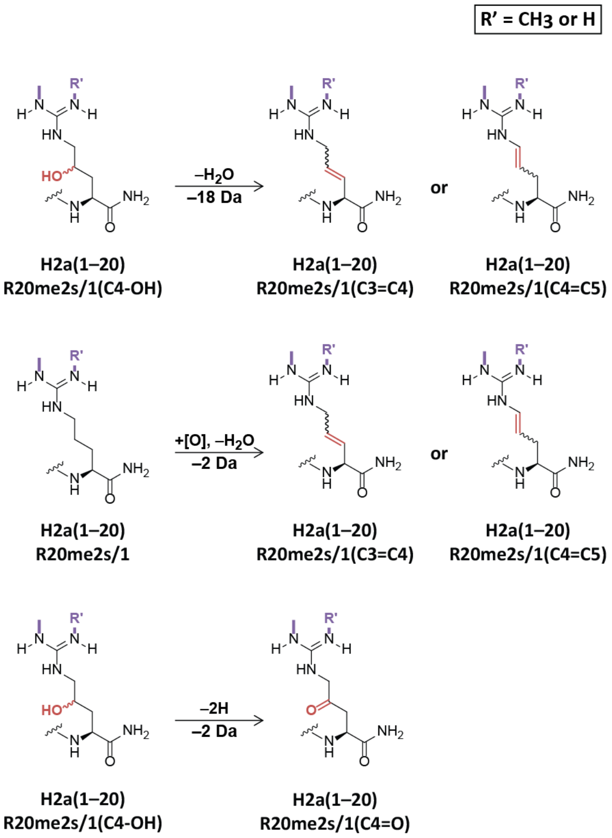

Supplementary Figure 12 Hydroxylation and demethylation of H2a(1–20)R20meX by KDM4E. LC–MS assays of KDM4E/H2a(1–20)R20meX **(a)** Representative LC–MS (120-minute incubation) of KDM4E with: **(from top to bottom)** H2a(1–20)R20me2s, H2a(1–20)R20me2a, and H2a(1–20)R20me1 and **(b)** 16-hour incubation of KDM4E with H2a(1–20)R20me1, showing both a +16 Da mass shift (hydroxylation), and −14 Da mass shifts (demethylation). Note the presence of some unassigned peaks, the presence of which varies depending on the substrate. Charge state of labelled ions: $[M + H]^{5+}$. n = 3 (independent assays). Y-axis: relative abundance (%). **(c)** Table of observed mass shifts together with the observed $[M + H]^{5+}$ and the monoisotopic mass this corresponds to. **(d)** Time-courses of the assays/species in **(a)/(c)**) for KDM4E incubations with **(top to bottom)** H2a(1–20)R20me2s, H2a(1–20)R20me2a, and H2a(1–20)R20me1. Conditions for **(a & c)** see **Supplementary Table 6**. Conditions for **(b)**: 4.74 µM KDM4E, 12 µM peptide, 200 µM 2OG, 100 µM sodium L-ascorbate, 10 µM $(NH_4)_2Fe(SO_4)_2$, and 50 mM HEPES (pH 7.5). **(e)** Some possible explanations for unassigned observed peaks on treatment of H2a(1–20)R20me2a/s and H2a(1–20)R20me1 with KDM4E; note there are other possibilities, e.g. cyclopropane instead of / in addition to alkene formation.[3]

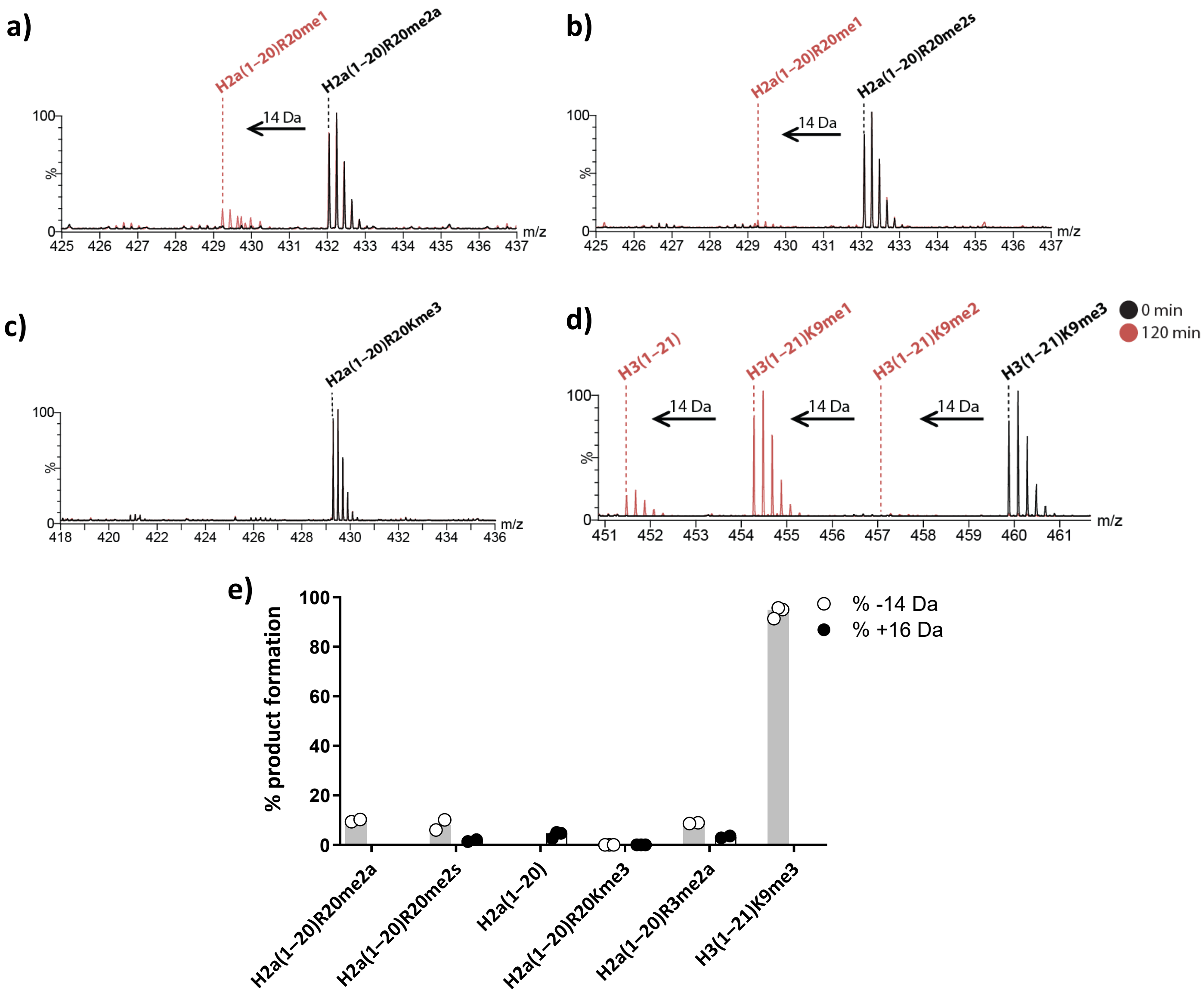

f)

Peptide	% product formation ± SD	% −14 Da ± SD	% +16 Da ± SD
H2a(1–20)R20me2a	9.82 ± 0.69	9.82 ± 0.69	/
H2a(1–20)R20me2s	9.83 ± 3.88	8.07 ± 2.87	1.76 ± 0.52
H2a(1–20)	4.11 ± 1.31	/	4.11 ± 1.31
H2a(1–20)R20Kme3	/	/	/
H2a(1–20)R3me2a	12.03 ± 0.52	8.79 ± 0.19	3.24 ± 0.55
H3(1–21)K9me3	94.02 ± 1.91	94.02 ± 1.91	/

Supplementary Figure 13 Reactivity of KDM4D with the H2a peptide panel. Representative LC–MS (120-minute incubation) of KDM4D at 10 µM with: **(a)** H2a(1–20)R20me2a, **(b)** H2a(1–20)R20me2s, **(c)** H2a(1–20)R20Kme3, and **(d)** H3(1–21)K9me3 showing −14 Da mass in all cases, except for **(c)** where no mass shift was observed. Charge state of labelled ions: $[M + H]^{5+}$. Y-axis: relative abundance (%). **(e)** Bar graph and **(f)** Table comparing % product formation and −14 Da and +16 Da mass shifts of KDM4D with H2a(1–20)R20me2a, H2a(1–20)R20me2s, H2a(1–20), H2a(1–20)R3me2a, H2a(1–20)R20Kme3, and H3(1–21)K9me3. n = 2/3 (independent assays). Conditions: 10 µM peptide, 200 µM 2OG, 100 µM sodium L-ascorbate, 10 µM $(NH_4)_2Fe(SO_4)_2$, and 50 mM HEPES (pH 7.5).

a)

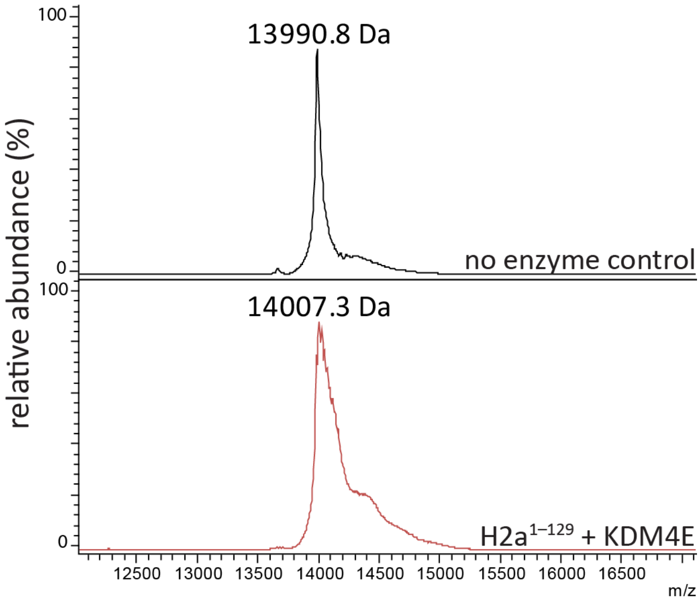

b) KDM4E + H2a(1–129)

Sequence coverage

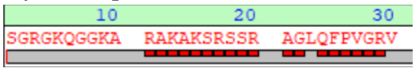

Spectrum view

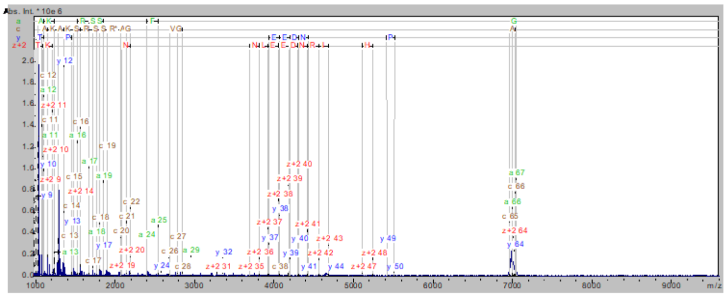

Ion table

Ion #	a	c	A.A	y	z+2	Ion #
1	60.044	105.066	Ser	13998.896	13983.885	129
2	117.066	162.087	Gly	13911.864	13896.853	128
3	273.167	318.188	Arg	13854.842	13839.832	127
4	330.188	375.210	Gly	13698.741	13683.730	126
5	458.283	503.305	Lys	13641.720	13626.709	125
6	586.342	631.363	Gln	13513.625	13498.614	124
7	643.363	688.385	Gly	13385.566	13370.555	123
8	700.385	745.406	Gly	13328.545	13313.534	122
9	828.480	873.501	Lys	13271.523	13256.513	121
10	899.517	944.538	Ala	13143.428	13128.418	120
11	1055.618	1100.640	Arg	13072.391	13057.380	119
12	1126.655	1171.677	Ala	12916.290	12901.279	118
13	1254.750	1299.772	Lys	12845.253	12830.242	117
14	1325.787	1370.809	Ala	12717.158	12702.147	116
15	1453.882	1498.904	Lys	12646.121	12631.110	115
16	1540.914	1585.936	Ser	12518.026	12503.015	114
17	1697.015	1742.037	Arg	12430.994	12415.983	113
18	1784.047	1829.069	Ser	12274.893	12259.882	112
19	1871.079	1916.101	Ser	12187.861	12172.850	111
20	2043.175	2088.197	Arg (+15.99)	12100.829	12085.818	110
21	2114.213	2159.234	Ala	11928.733	11913.722	109
22	2171.234	2216.255	Gly	11857.696	11842.685	108
23	2284.318	2329.340	Leu	11800.674	11785.663	107
24	2412.377	2457.398	Gln	11687.590	11672.579	106
25	2559.445	2604.467	Phe	11559.532	11544.521	105
26	2656.498	2701.519	Pro	11412.463	11397.452	104
27	2755.566	2800.588	Val	11315.410	11300.400	103
28	2812.588	2857.609	Gly	11216.342	11201.331	102
29	2968.689	3013.710	Arg	11159.321	11144.310	101

c) H2a(1–129)

Sequence coverage

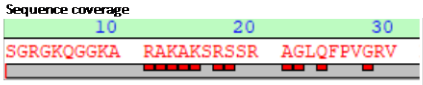

Spectrum view

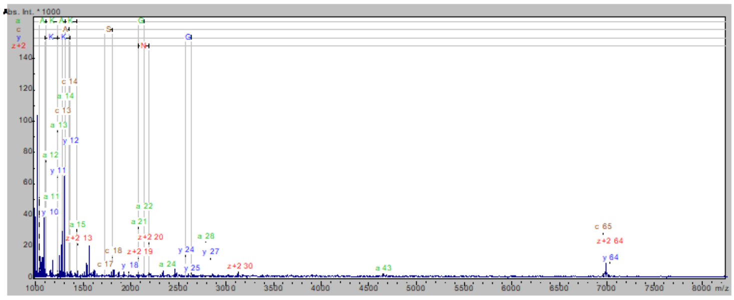

Ion table

Ion #	a	c	A.A	y	z+2	Ion #
1	60.044	105.066	Ser	13982.901	13967.890	129
2	117.066	162.087	Gly	13895.869	13880.858	128
3	273.167	318.188	Arg	13838.847	13823.837	127
4	330.188	375.210	Gly	13682.746	13667.735	126
5	458.283	503.305	Lys	13625.725	13610.714	125
6	586.342	631.363	Gln	13497.630	13482.619	124
7	643.363	688.385	Gly	13369.571	13354.560	123
8	700.385	745.406	Gly	13312.550	13297.539	122
9	828.480	873.501	Lys	13255.528	13240.518	121
10	899.517	944.538	Ala	13127.433	13112.423	120
11	1055.618	1100.640	Arg	13056.396	13041.385	119
12	1126.655	1171.677	Ala	12900.295	12885.284	118
13	1254.750	1299.772	Lys	12829.258	12814.247	117
14	1325.787	1370.809	Ala	12701.163	12686.152	116
15	1453.882	1498.904	Lys	12630.126	12615.115	115
16	1540.914	1585.936	Ser	12502.031	12487.020	114
17	1697.015	1742.037	Arg	12414.999	12399.988	113
18	1784.047	1829.069	Ser	12258.898	12243.887	112
19	1871.079	1916.101	Ser	12171.866	12156.855	111
20	2027.181	2072.202	Arg	12084.834	12069.823	110
21	2098.218	2143.239	Ala	11928.733	11913.722	109
22	2155.239	2200.261	Gly	11857.696	11842.685	108
23	2268.323	2313.345	Leu	11800.674	11785.663	107
24	2396.382	2441.403	Gln	11687.590	11672.579	106
25	2543.450	2588.472	Phe	11559.532	11544.521	105
26	2640.503	2685.524	Pro	11412.463	11397.452	104
27	2739.571	2784.593	Val	11315.410	11300.400	103
28	2796.593	2841.614	Gly	11216.342	11201.331	102
29	2952.694	2997.715	Arg	11159.321	11144.310	101

Supplementary Figure 14 Evidence KDM4E catalyses hydroxylation of full-length histone H2a at R20. MS analysis of recombinant full-length histone H2a(1–129) treated with KDM4E. (a) Representative MALDI–TOF MS of H2a(1–129) incubated for 16 hours with KDM4E in red compared with no enzyme control in black indicating evidence for possible modification. Charge state of labelled ions: $[MH]^+$. (b) MALDI Top-Down Sequencing (TDS) data for product of reaction between histone H2a(1–129) and KDM4E identifying Arg-20 as the residue with the +15.99 Da modification. (c) MALDI TDS on intact histone H2a(1–129) showing no evidence for modification. MALDI TDS results were analysed using Biotools software. Conditions: 16 µM KDM4E, 35 µM H2a(1–129), 100 µM 2OG, 100 µM sodium L-ascorbate, 10 µM $(NH_4)_2Fe(SO_4)_2$, and 50 mM HEPES (pH 7.5).

a)

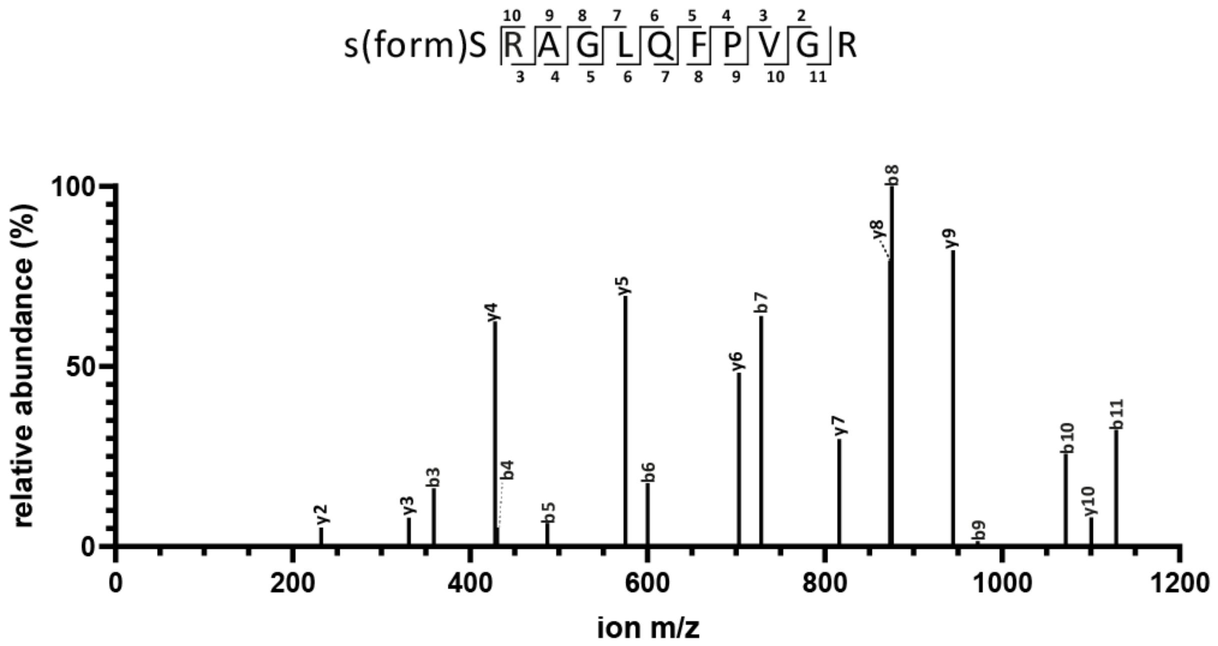

b)

#	b	Seq	y	#
1	116.03	S(form = +42.01)		12
2	203.07	S	1203.66	11
3	375.38	R (C4-OH = +16.00)	1116.89	10
4	446.44	A	944.69	9
5	503.23	G	873.63	8
6	616.57	L	816.98	7
7	744.60	Q	703.59	6
8	891.73	F	575.59	5
9	988.49	P	428.44	4
10	1088.01	V	331.45	3
11	1144.86	G	232.24	2
12		R	175.12	1

c)

#	b	Seq	y	#
1	116.03	S(form = +42.01)		12
2	203.18	S	1187.66	11
3	359.15	R	1100.74	10
4	430.50	A	944.64	9
5	487.19	G	873.59	8
6	600.43	L	816.54	7
7	728.46	Q	703.44	6
8	875.53	F	575.43	5
9	972.71	P	428.32	4
10	1071.69	V	331.17	3
11	1128.71	G	232.13	2
12		R	175.12	1

Supplementary Figure 15 Evidence KDM4E catalyses hydroxylation of full-length calf thymus histone H2a at R20. (a) LC–MS/MS fragment analysis of untreated H2a protein from calf thymus – no enzyme control was analysed on Orbitrap Elite MS, fragmented using collision-induced dissociation (CID), and the results were analysed using PEAKS software. No hydroxylation was detected in the unmodified peptide fragment S(+27.99)SRAGLQFPVGR having a −10logP score of 50.3**.** The +28 Da mass shift on the N-terminal Ser is due to N-formylation caused by formic acid quenching the tryptic digest during sample processing. This is the negative control for the LC–MS/MS analysis of KDM4E treated calf histone H2a **(Figure 5b)**. Ion tables to compliment LC–MS/MS fragment analysis of KDM4E-treated **(b)** and untreated H2a protein from calf thymus in **(c).** Conditions: 9 µM KDM4E, 0.2 µg.µL^{-1} calf thymus histone protein, 200 µM 2OG, 100 µM sodium L-ascorbate, 10 µM $(NH_4)_2Fe(SO_4)_2$, and 50 mM HEPES (pH 7.5).

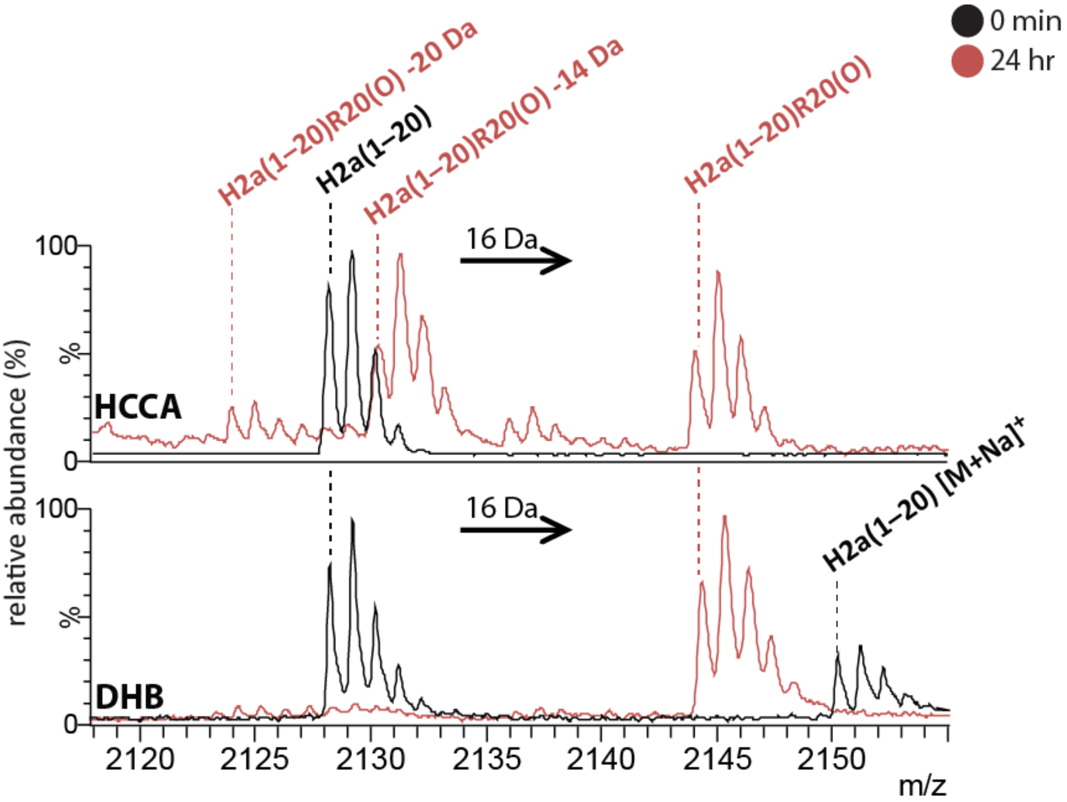

Supplementary Figure 16 An artefact is formed when the product of H2a(1–20) and KDM4E is analysed using α-cyano-4-hydroxycinnamic acid (HCCA) and MALDI–TOF MS. Representative MALDI–TOF MS following 24-hour incubation of KDM4E with H2a(1–20). The red spectra are from the same sample treated with HCCA MALDI matrix (upper) or 2,5-dihydroxybenzoic acid (2,5-DHB) MALDI matrix (lower). +16 Da shifts in mass corresponding to the hydroxylated product of the H2a(1–20) and KDM4E reaction [H2a(1–20)R20(O)] are present in both spectra. The H2a(1–20) starting material spectra are in black. An additional −14 Da peak (relative to the hydroxylated product) is present in the sample treated with HCCA, but not with the 2,5-DHB-treated sample. n = 2 (independent assays). Charge state of labelled ions: $[MH]^+$. Conditions: 9 µM KDM4E, 10 µM peptide, 200 µM 2OG, 100 µM sodium L-ascorbate, 10 µM $(NH_4)_2Fe(SO_4)_2$, and 50 mM HEPES (pH 7.5).

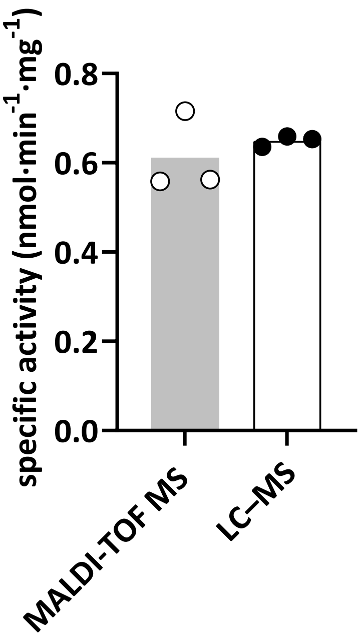

Supplementary Figure 17 Comparison of specific hydroxylation activities of KDM4E with H2a(1–20) when analysed by MALDI-TOF MS or LC–MS. Comparison of specific activities for KDM4E catalysed hydroxylation of H2a(1–20) when analysed with MALDI-TOF MS or LC–MS (0.61 nmol·min^{-1}·mg^{-1} SD: 0.073 and 0.65 nmol·min^{-1}·mg^{-1} SD: 0.01 respectively). n = 3 (independent assays). See **Supplementary Table 6** for assay conditions.

		1	2	3	4	5	6	7	8	9	10	11	12	13	14	15	16	17	18	19	20	21	22	23	24	25
				Ci						Ac																
	Ac	P		Me		Ac				Me		Me		Ac		Ac	HAR domain									
H2a	*N*-	S	G	R	G	K	Q	G	G	K	A	R	A	K	A	K	T	R	S	S	R	A	G	L	Q	F

Supplementary Table 1 Reported post-translational modifications (PTMs) on the N-terminus of histone H2a.[4,5] Ac = acetylation, P = phosphorylation, Me = methylation, Ci = citrullination, HAR = H2a repression domain

Protein	N-			substrate			C-	Ref
H1.4	24	A	R	**K**	S	A	28	6
H2a	18	S	S	**K**	A	G	22	
H3	7	A	R	**K**	S	T	11	7
H2a	1	S	G	**R**	G	K	5	
	18	S	S	**R**	A	G	22	
H3	1		A	**R**	T	K	4	1,2,8
	6	T	A	**R**	K	S	10	1,8
	7	A	R	**R**	S	T	11	1,8
	24	A	A	**R**	K	S	28	1,8
H4	1	S	G	**R**	G	K	5	1,8
hnRNPK	297	G	G	**R**	G	G	231	8
BRCA1	608	K	N	**R**	L	R	612	8
FMR1	542	R	G	**R**	G	G	544	8

Sequence Logo

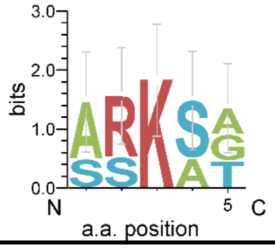

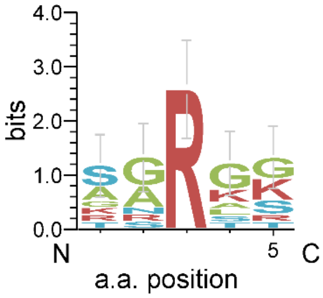

Supplementary Table 2 Comparison of sequences surrounding lysine and arginine demethylation substrates of KDM4E. Amino acid residues are coded in colours corresponding to the properties of their side chains. **Green**: hydrophobic: aliphatic, **blue**: hydrophilic: polar uncharged, and **red**: hydrophilic: basic. Underlined residues denote variants of the natural histone substrate of KDM4E. The consensus logo was created using Web Logo 3.[9]

Name	Common Name	Variant	Accession no	N-					HAR domain			20			C-
H. sapiens	Human	H2a	B2R5B3	13	K	A	K	T	R	S	S	R	A	G	22
		H2aC1	Q96QV6	13	K	S	K	S	R	S	S	R	A	G	22
M. musculus	Mouse	H2a	B2RWH3	13	K	A	K	S	R	S	S	R	A	G	22
K. laevis	African clawed frog	H2a type 1	P06897	13	K	A	K	T	R	S	S	R	A	G	22
D. melanogaster	Fruit fly	His2A	P84051	12	K	A	K	S	R	S	N	R	A	G	21
C. elegans		H2a	P09588	14	K	A	K	S	R	S	S	R	A	G	23
S. pombe	Fission yeast	H2a-beta	P04910	14	S	A	Q	S	R	S	A	K	A	G	23
S. cerevisiae	Baker's yeast	H2a	A6ZYH9	14	A	S	Q	S	R	S	A	K	A	G	23
		H2a.Z	Q12692	20	R	S	Q	S	S	S	A	R	A	G	30

Supplementary Table 3 Comparison of HAR domain sequences of histone H2a across species. Residues are colour coded according to side chain properties. **Green**: hydrophobic: aliphatic, **blue**: hydrophilic: polar uncharged, and **red**: hydrophilic: basic. Residues of the H2a repression (HAR) domain are marked with a red line. Sequence data were sourced from Uniprot[10] and compared using BLAST+.[11]

Enzyme	Sequence	Expressed in	SDS-PAGE	Calculated MW (Da)	Observed MW (Da)	MS	Reference
KDM4A*	1–1,064	Sf9 cells					
KDM4A	1–359		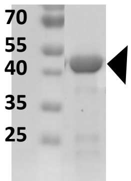				12
KDM4B	1–359		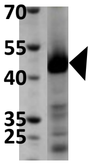	44295	44295	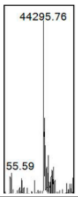	
KDM4C	1–359	*E. coli*					
KDM4D	1–358		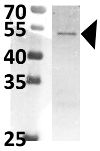	43752	43760	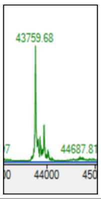	13
KDM4E	1–337		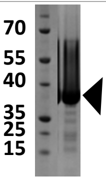	39818	39819	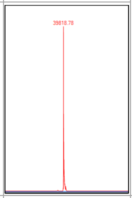	
KDM5A	13–87, GGGG, 354–744		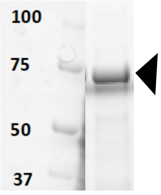				14
KDM5B	1–822		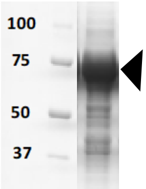				15
KDM5C	1–765	Sf9 cells	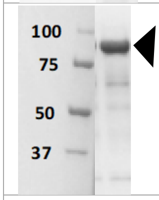				16
KDM5D	1–775		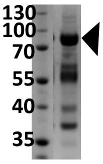	92407	92447	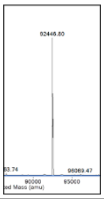	17
FDH (*Pseudomonas*)	1-399	*E. coli*					18

Supplementary Table 4 Summary of constructs, expression systems used and characterisation of purified enzymes. SDS-PAGE analysis demonstrating purities of enzymes used in our work. Deconvoluted LC–MS analyses for selected enzymes**.** *KDM4A$^{1–1,064}$ was from Active Motif (Catalogue No: 31457). Table adapted from Figure S1[1] and Table S1[1] under the CC BY 4.0 license terms.

peptide	sequence	Monoisotopic Mass (Da)	[M + H]$^{+}$	[M + H]$^{5+}$	Source
H2a(1–20)R3me2a (AB)	SGR[me2a]GKQGGKARAKAKTRSSR-NH-Ahx-K-Biotin	2580.91	2581.91	517.18	AltaBioscience
H2a(1–20)R3me2s (AB)	SGR[me2s]GKQGGKARAKAKTRSSR-NH-Ahx-K-Biotin	2580.91	2581.91	517.18	AltaBioscience
H2a(1–20)R3me1 (AB)	SGR[me1]GKQGGKARAKAKTRSSR-NH-Ahx-K-Biotin	2566.90	2567.90	514.38	AltaBioscience
H2a(1–20) (AB)	SGRGKQGGKARAKAKTRSSR-NH-Ahx-K-Biotin	2552.88	2553.88	511.58	AltaBioscience
H2a(1–20)Ci3 (AB)	SGR[Ci]GKQGGKARAKAKTRSSR-NH-Ahx-K-Biotin	2553.87	2554.87	511.77	AltaBioscience
H2a(1–20)R3me2a	Ac-SGR[me2a]GKQGGKARAKAKTRSSR	2155.25	2156.25	432.05	In house
H2a(1–20)	Ac-SGRGKQGGKARAKAKTRSSR	2127.22	2128.22	426.44	In house/GL Biochem
H2a(1–20)-OH*	Ac-SGRGKQGGKARAKAKTRSSR-OH	2128.20	2129.20	426.64	GL Biochem
H2a(1–20)R20A	Ac-SGRGKQGGKARAKAKTRSSA	2042.16	2043.16	409.43	In house
H2a(1–20)R20K	Ac-SGRGKQGGKARAKAKTRSSK	2099.21	2100.21	420.84	In house
H2a(1–20)R20Kme3	Ac-SGRGKQGGKARAKAKTRSSK[me3]	2141.26	2142.26	429.25	In house
H2a(1–20)R3A	Ac-SGAGKQGGKARAKAKTRSSR	2042.16	2043.16	409.43	In house
H2a(1–21)	Ac-SGRGKQGGKARAKAKTRSSRA	2198.26	2199.26	440.65	In house/GL Biochem
H2a(1–25)	Ac-SGRGKQGGKARAKAKTRSSRAGLQF	2643.49	2644.49	529.70	In house
H2a(10–29)	Ac-AKTRSSRAGLQFPVGRVHRL	2276.31	2277.31	456.26	In house
H2a(1–20)R20me2s	Ac-SGRGKQGGKARAKAKTRSSR[me2s]	2155.25	2156.25	432.05	In house
H2a(1–20)R20me2a	Ac-SGRGKQGGKARAKAKTRSSR[me2a]	2155.25	2156.25	432.05	In house
H2a(1–20)R20me1	Ac-SGRGKQGGKARAKAKTRSSR[me1]	2141.24	2142.24	429.25	In house
H3(1–21)R2me2a	AR[me2a]TKQTARKSTGGKAPRKQLA	2280.36	2281.36	457.07	In house
H3(1–21)K9me3	ARTKQTARK[me3]STGGKAPRKQLA	2294.38	2295.38	459.88	GL Biochem
H3(1–21)K9Rme2a	ARTKQTARR[me2a]STGGKAPRKQLA	2308.37	2309.37	462.67	In house
H3(1–21)K9R	ARTKQTARRSTGGKAPRKQLA	2280.34	2281.34	457.07	In house
H4(1-21)R3Kme3	Ac-SGK[me3]GKGGKGLGKGGAKRHRKV	2146.29	2147.29	430.26	In house
H4(1-21)R3K	Ac-SGKGKGGKGLGKGGAKRHRKV	2104.25	2105.25	421.85	In house
H4(1-21)	Ac-SGRGKGGKGLGKGGAKRHRKV	2132.25	2133.25	427.45	In house
H4(1-21)R3me2a	Ac-SGR[me2a]GKGGKGLGKGGAKRHRKV	2160.28	2161.28	433.06	In house

Supplementary Table 5 Peptides used. Peptides were synthesised as C-terminal amides except for *H2a(1–20)-OH (which was synthesised with a C-terminal carboxylic acid) and peptides with a C-terminal N^{ε}-(D-biotin)-L-lysine-amide (NH-Ahx-K-biotin) group which was linked via an aminohexanoic acid unit. Peptides were synthesised using a Liberty Blue machine (CEM), purified to >95% by HPLC (C18 column) and quantified by NMR. Purity and mass were analysed by LC–MS. Nomenclature: H2a/H3 represent the histone; the numbers correspond to histone residue numbers. K/R[X] corresponds to the modified lysine or arginine. Modifications are denoted as me1, me2, me3, for mono-, di-, and tri-methylations, respectively; me2a is asymmetric di-methyl arginine; me2s is symmetric di-methyl arginine; Ci is citrulline; and Ac represents acetylation.

Enzyme	Cognate substrate	[Enzyme] (mM)	[Peptide] (mM)	[2OG] (mM)	[Ascorbate] (mM)	[$(NH_4)_2Fe(SO_4)_2$] (mM)	[TCEP] (mM)	Buffer pH7.5	
								[HEPES] (mM)	[NaCl] (mM)
KDM4A	H3(1–21)K9me3 [19]	2	10	100	100	10	/	50	/
KDM4B	H3(1–21)K9me3 [19]	2	10	100	100	10	/	50	/
KDM4C	H3(1–21)K9me3 [19]	2	10	100	100	10	/	50	/
KDM4D	H3(1–21)K9me3 [19]	2	10	100	100	10	/	50	/
KDM4E	H3(1–21)K9me3 [7]	2	10	100	100	10	/	50	/
KDM5A	H3(1–21)K4me3 [20,21]	2	10	100	100	10	/	50	/
KDM5B	H3(1–21)K4me3 [20,21]	2	10	100	100	10	/	50	/
KDM5C	H3(1–21)K4me3 [20,21]	2	10	100	100	10	/	50	/
KDM5D	H3(1–21)K4me3 [21,22]	2	10	100	100	10	5	50	50

Supplementary Table 6 Summary of MS assay conditions for substrate screening. These conditions enabled efficient turnover of established substrates. Reactions were carried out at 37 ^{o}C unless otherwise stated. Table adapted from Table S3[1] under the CC BY 4.0 license terms.

Supplementary References

1. Bonnici, J. *et al.* The catalytic domains of all human KDM5 JmjC demethylases catalyse N-methyl arginine demethylation. *FEBS Lett* **597**, 933–946 (2023).
2. Bonnici, J., Tumber, A., Kawamura, A. & Schofield, C. J. Inhibitors of both the N-methyl lysyl- and arginyl-demethylase activities of the JmjC oxygenases. *Philosophical Transactions of the Royal Society B: Biological Sciences* **373**, (2018).
3. Ushimaru, R. *et al.* Mechanistic Analysis of Stereodivergent Nitroalkane Cyclopropanation Catalyzed by Nonheme Iron Enzymes. *J Am Chem Soc* **145**, 24210–24217 (2023).
4. Huang, H., Sabari, B. R., Garcia, B. A., David Allis, C. & Zhao, Y. SnapShot: Histone modifications. *Cell* vol. 159 458 Preprint at https://doi.org/10.1016/j.cell.2014.09.037 (2014).
5. Corujo, D. & Buschbeck, M. Post-Translational Modifications of H2A Histone Variants and Their Role in Cancer. *Cancers (Basel)* **10**, 59 (2018).
6. Walport, L. J. *et al.* Mechanistic and structural studies of KDM-catalysed demethylation of histone 1 isotype 4 at lysine 26. *FEBS Lett* **592**, (2018).
7. Hillringhaus, L. *et al.* Structural and evolutionary basis for the dual substrate selectivity of human KDM4 histone demethylase family. *J Biol Chem* **286**, 41616–41625 (2011).
8. Walport, L. J. *et al.* Arginine demethylation is catalysed by a subset of JmjC histone lysine demethylases. *Nat Commun* **7**, 11974 (2016).
9. Crooks, G. & Fabros, M. WebLogo 3.7. Preprint at (2022).
10. Consortium, T. U. UniProt: the Universal Protein Knowledgebase in 2023. *Nucleic Acids Res* **51**, D523–D531 (2023).
11. Camacho, C. *et al.* BLAST+: architecture and applications. *BMC Bioinformatics* **10**, 421 (2009).
12. Ng, S. S. *et al.* Crystal structures of histone demethylase JMJD2A reveal basis for substrate specificity. *Nature* **448**, 87–91 (2007).
13. Rose, N. R. *et al.* Inhibitor Scaffolds for 2-Oxoglutarate-Dependent Histone Lysine Demethylases. *J Med Chem* **51**, 7053–7056 (2008).
14. Tumber, A. *et al.* Potent and Selective KDM5 Inhibitor Stops Cellular Demethylation of H3K4me3 at Transcription Start Sites and Proliferation of MM1S Myeloma Cells. *Cell Chem Biol* **24**, 371–380 (2017).
15. Johansson, C. *et al.* Structural analysis of human KDM5B guides histone demethylase inhibitor development. *Nat Chem Biol* **12**, 539–545 (2016).
16. Rose, N. R. *et al.* Plant growth regulator daminozide is a selective inhibitor of human KDM2/7 histone demethylases. *J Med Chem* **55**, 6639–6643 (2012).

17. Belle, R. Histone Lysine and DNA Methylation: Dynamic Marks in the Chromatin. (Oxford, 2019).

18. Lizcano, J. M., Unzeta, M. & Tipton, K. F. A spectrophotometric method for determining the oxidative deamination of methylamine by the amine oxidases. *Anal Biochem* **286**, 75–79 (2000).

19. Whetstine, J. R. *et al.* Reversal of Histone Lysine Trimethylation by the JMJD2 Family of Histone Demethylases. *Cell* **125**, 467–481 (2006).

20. Christensen, J. *et al.* RBP2 Belongs to a Family of Demethylases, Specific for Tri-and Dimethylated Lysine 4 on Histone 3. *Cell* **128**, 1063–1076 (2007).

21. Iwase, S. *et al.* The X-Linked Mental Retardation Gene SMCX/JARID1C Defines a Family of Histone H3 Lysine 4 Demethylases. *Cell* **128**, 1077–1088 (2007).

22. Lee, M. G., Norman, J., Shilatifard, A. & Shiekhattar, R. Physical and Functional Association of a Trimethyl H3K4 Demethylase and Ring6a/MBLR, a Polycomb-like Protein. *Cell* **128**, 877–887 (2007).
