# Supplementary material for: JmjC catalysed histone H2a N-methyl arginine demethylation and C4-arginine hydroxylation reveals importance of sequence-reactivity relationships
Source: Commun Biol. 2024 Nov 27;7:1583. doi: 10.1038/s42003-024-07183-5 (PMC11603075; doi:10.1038/s42003-024-07183-5)
Supplement: Supplementary file 4 — Reporting Summary [file 42003_2024_7183_MOESM4_ESM.pdf]

# nature portfolio

Corresponding author(s): Christopher J. Schofield

Last updated by author(s): Jul 17, 2024

# Reporting Summary

Nature Portfolio wishes to improve the reproducibility of the work that we publish. This form provides structure for consistency and transparency in reporting. For further information on Nature Portfolio policies, see our Editorial Policies and the Editorial Policy Checklist.

## Statistics

For all statistical analyses, confirm that the following items are present in the figure legend, table legend, main text, or Methods section.

| n/a | Confirmed | |
|---|---|---|
| ☐ | ☒ | The exact sample size (*n*) for each experimental group/condition, given as a discrete number and unit of measurement |
| ☒ | ☐ | A statement on whether measurements were taken from distinct samples or whether the same sample was measured repeatedly |
| ☒ | ☐ | The statistical test(s) used AND whether they are one- or two-sided<br>*Only common tests should be described solely by name; describe more complex techniques in the Methods section.* |
| ☒ | ☐ | A description of all covariates tested |
| ☒ | ☐ | A description of any assumptions or corrections, such as tests of normality and adjustment for multiple comparisons |
| ☐ | ☒ | A full description of the statistical parameters including central tendency (e.g. means) or other basic estimates (e.g. regression coefficient) AND variation (e.g. standard deviation) or associated estimates of uncertainty (e.g. confidence intervals) |
| ☒ | ☐ | For null hypothesis testing, the test statistic (e.g. *F*, *t*, *r*) with confidence intervals, effect sizes, degrees of freedom and *P* value noted<br>*Give P values as exact values whenever suitable.* |
| ☒ | ☐ | For Bayesian analysis, information on the choice of priors and Markov chain Monte Carlo settings |
| ☒ | ☐ | For hierarchical and complex designs, identification of the appropriate level for tests and full reporting of outcomes |
| ☒ | ☐ | Estimates of effect sizes (e.g. Cohen's *d*, Pearson's *r*), indicating how they were calculated |

*Our web collection on statistics for biologists contains articles on many of the points above.*

## Software and code

Policy information about availability of computer code

| | |
|---|---|
| Data collection | MassHunter workstation (Agilent Technologies), TopSpin 4 (Bruker), microplate Reader Control (BMG LABTECH), Xcalibur software v 4.1.31.9 (Thermo Fisher Scientific), FlexControl v3.4 (Bruker), TopSpin 3.2 software (Bruker), MassLynx 4.1 software (Waters), PHERastar - Control v 5.41 (BMG) |
| Data analysis | Prism 10.1.2 (GraphPad), MestReNova 14.3.2 (Mestrelab Research), MassHunter Qualitative Analysis B.07.00 (Agilent Technologies) GraphPad Prism5 version 5.04, Xcalibur software v 4.1.31.9 (Thermo Fisher Scientific), Peaks 8.5 (Bioinformatics Solutions Inc), FlexAnalysis v3.4 (Bruker), Biotools (Bruker), TopSpin 3.2 software (Bruker), MassLynx 4.1 software (Waters), PHERastar - MARS (data analysis) v 3.32 |

For manuscripts utilizing custom algorithms or software that are central to the research but not yet described in published literature, software must be made available to editors and reviewers. We strongly encourage code deposition in a community repository (e.g. GitHub). See the Nature Portfolio guidelines for submitting code & software for further information.

## Data

Policy information about availability of data

All manuscripts must include a data availability statement. This statement should provide the following information, where applicable:

- Accession codes, unique identifiers, or web links for publicly available datasets
- A description of any restrictions on data availability
- For clinical datasets or third party data, please ensure that the statement adheres to our policy

The authors declare that the data that supports the findings of this study are available within the paper [and its supplementary files]. Source data for all figures and tables are available within the supplementary data file.

## Human research participants

Policy information about studies involving human research participants and Sex and Gender in Research.

| | |
|---|---|
| Reporting on sex and gender | Not applicable |
| Population characteristics | Not applicable |
| Recruitment | Not applicable |
| Ethics oversight | Not applicable |

Note that full information on the approval of the study protocol must also be provided in the manuscript.

# Field-specific reporting

Please select the one below that is the best fit for your research. If you are not sure, read the appropriate sections before making your selection.

☒ Life sciences ☐ Behavioural & social sciences ☐ Ecological, evolutionary & environmental sciences

For a reference copy of the document with all sections, see nature.com/documents/nr-reporting-summary-flat.pdf

# Life sciences study design

All studies must disclose on these points even when the disclosure is negative.

| | |
|---|---|
| Sample size | Sample size are noted in the methods / figure legends. Biological replicate of three, with three technical repeats were chosen for data generation for biochemical work on isolated recombinant enzyme |
| Data exclusions | no data were excluded from the analyses |
| Replication | all attempts at replication were successful<br>where relevant, n = 3/2, independent assays were carried out for each experiment and in some cases, each experiment was carried out three times. Mean and standard deviations are provided where appropriate, indicating high reproducibility |
| Randomization | Not applicable |
| Blinding | Not applicable |

# Reporting for specific materials, systems and methods

We require information from authors about some types of materials, experimental systems and methods used in many studies. Here, indicate whether each material, system or method listed is relevant to your study. If you are not sure if a list item applies to your research, read the appropriate section before selecting a response.

## Materials & experimental systems

| n/a | Involved in the study |
| --- | --- |
| ☒ | ☐ Antibodies |
| ☒ | ☐ Eukaryotic cell lines |
| ☒ | ☐ Palaeontology and archaeology |
| ☒ | ☐ Animals and other organisms |
| ☒ | ☐ Clinical data |
| ☒ | ☐ Dual use research of concern |

## Methods

| n/a | Involved in the study |
| --- | --- |
| ☒ | ☐ ChIP-seq |
| ☒ | ☐ Flow cytometry |
| ☒ | ☐ MRI-based neuroimaging |
